# Supplementary material for: Chemical linkers switch triglycerol detergents from bacterial protein purification to mild antibiotic amplification
Source: Commun Chem. 2025 Mar 8;8:70. doi: 10.1038/s42004-025-01477-3 (PMC11890857; doi:10.1038/s42004-025-01477-3)
Supplement: Supplementary file 2 — supplementary information [file 42004_2025_1477_MOESM2_ESM.pdf]

Supplementary Information

Chemical linkers switch triglycerol detergents from bacterial protein purification to mild antibiotic amplification

Abhishek Kumar Singh,[‡,a*] Marc Seewald,[‡,b] Boris Schade,[a] Christian Zoiester,[a] Rainer Haag,[a] Leonhard Hagen Urner[b*]

Table of contents

1. Supplementary Figures 2
2. Supplementary Tables 21
3. Detergent Synthesis 23
4. Supplementary References 34

1. Supplementary Figures

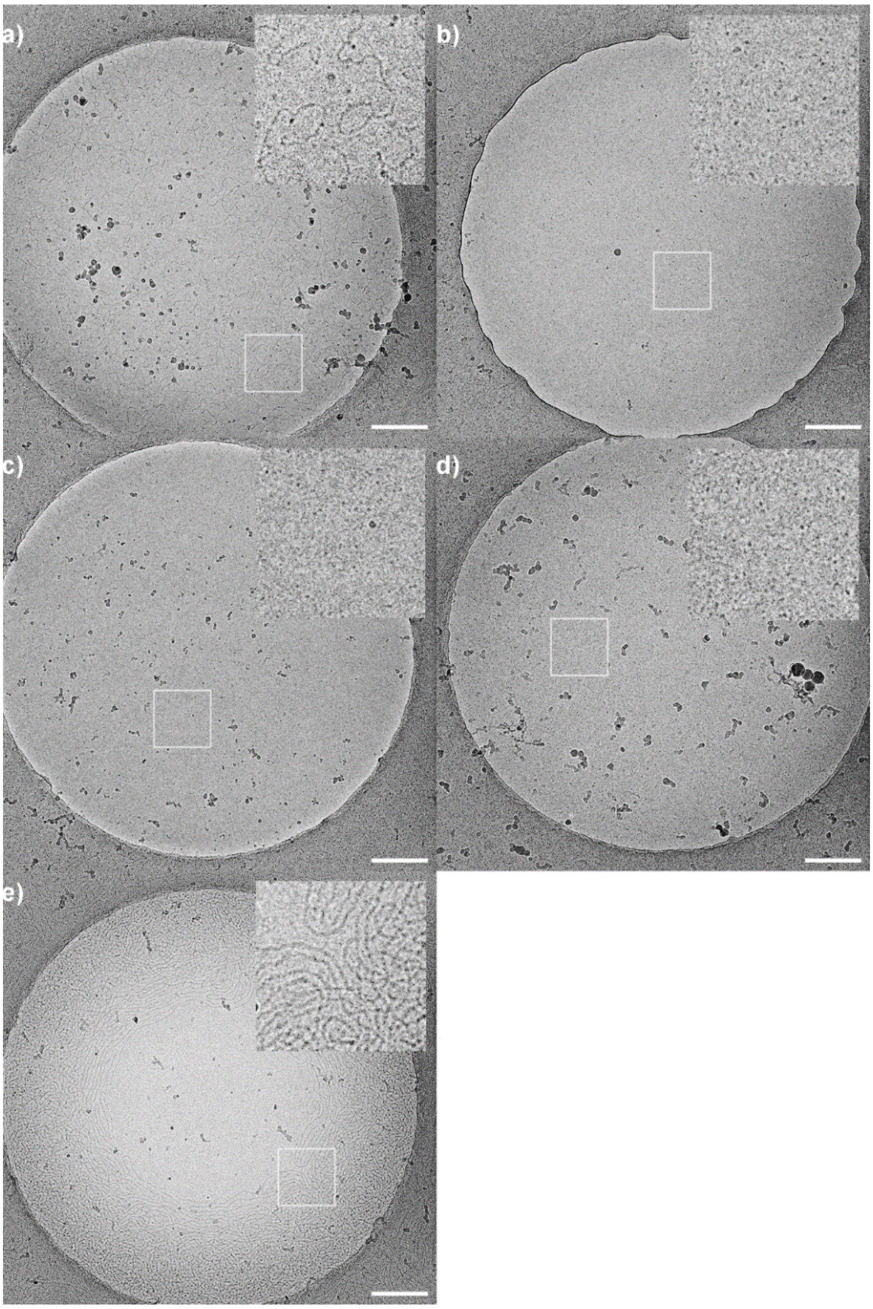

Supplementary Figure 1. Investigation of detergent aggregates by cryoelectron microscopy. Cryogenic electron microscopy (Cryo-EM) obtained from triglycerol detergents in water at a concentration of 2.5 mg/mL, i.e., a) DTG-thioether-C12 (worm-like micelles); b) DTG-ether-C12 (globular micelles); c) DTG-tirazole-C12 (globular micelles); d) DTG-amide-C12 (globular micelles); e) LTG-thioether-C12 (worm-like micelles). The length of the white scale bars corresponds to a length of 200 nm on the grids.

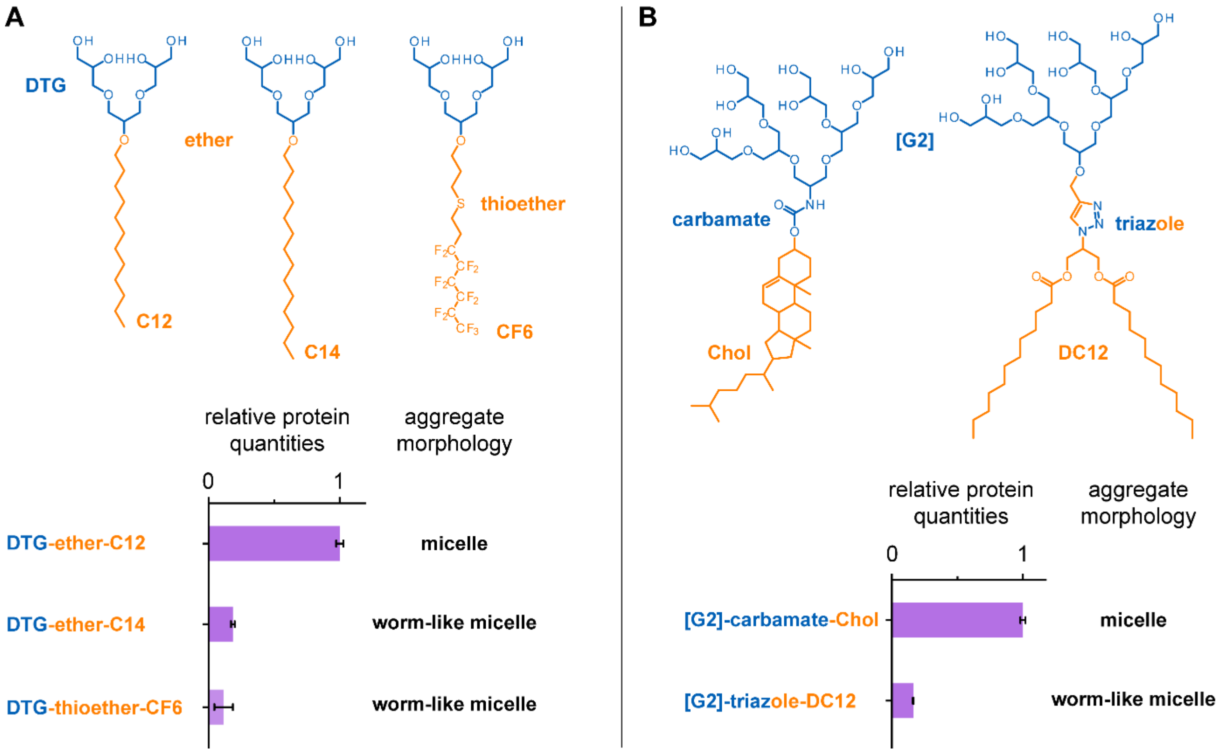

Supplementary Figure 2. Comparison of relative protein yields of AqpZ-GFP and aggregate morphologies. Bar charts (purple bars) visualize relative protein quantities of AqpZ-GFP obtained upon extraction and affinity purification with A) DTG detergents and B) second-generation DTG detergents (±SD, n = 3). Detergents that form worm-like micelles gave lower protein yields, regardless of the structure of the head group and tail. Relative protein quantities shown in A) were taken from Ref.[1] and relative protein quantities shown in B) were taken from Ref.[2] Source data are provided as Supplementary Data file.

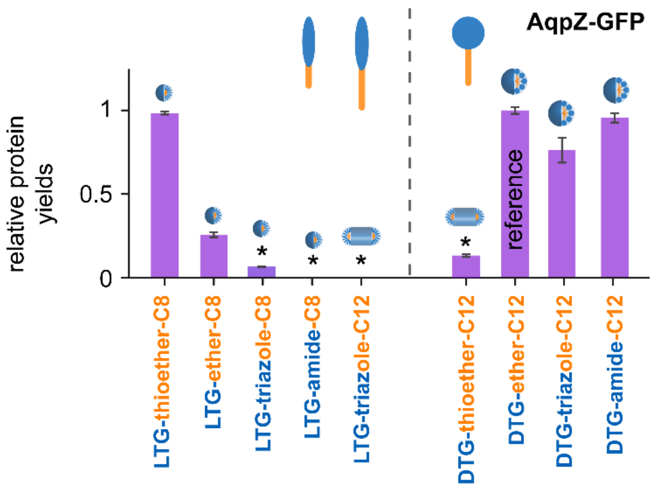

Supplementary Figure 3. Supramolecular chemistry affects protein purification. A) Bar chart (purple bars) showing relative protein yields (±SE) obtained upon extraction and IMAC purification of bacterial AqpZ-GFP from *E. coli* with different detergents and standard deviation from two independent repeats (n=2). The aggregate morphologies obtained from the detergents above cmc is indicated by schematics of globular micelles or worm-like micelles. Source data are provided as Supplementary Data file.

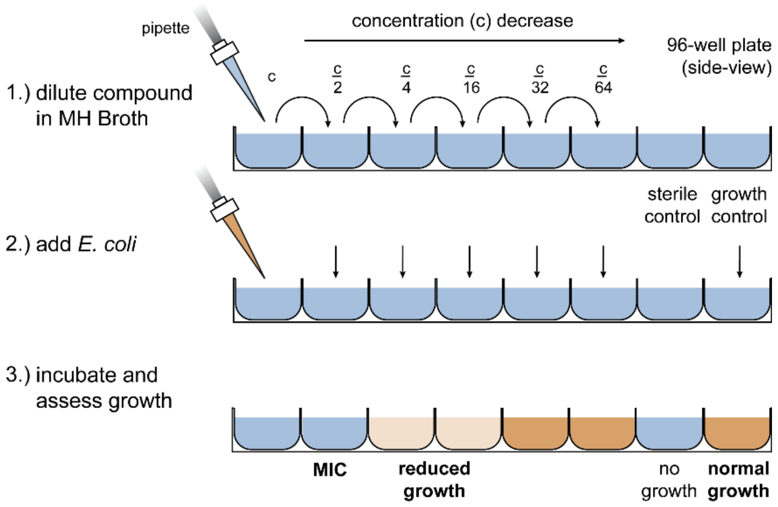

Supplementary Figure 4. Schematic of the Broth microdillution assay used for MIC determination. After the preparation of a concentration series of the compounds to be tested, *E. coli* suspensions are added to the wells. Growth assessment after incubation at 37 °C delivers the MIC values. Bacterial growth is assessed in comparison to a steril control and an untreated growth control.

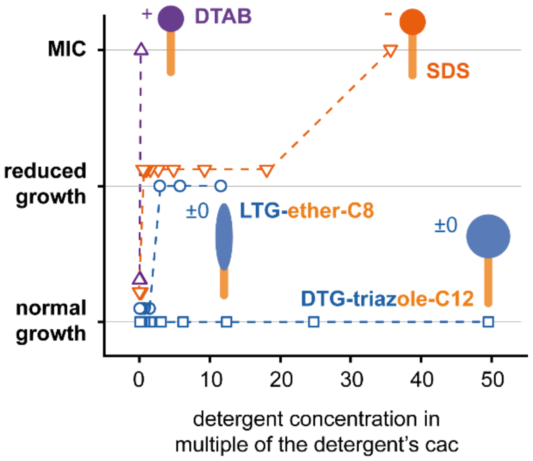

Supplementary Figure 5. Schematic showing MIC, reduced growth and normal growth of *E. coli* K12 MG1655 observed in a broth dillution assay with different detergents at different detergent concentrations. Trends were highlighted with dashed lines. The different detergents include DTAB (purple triangles), SDS (orange triangles), LTG-ether-C8 (blue circles), DTG-triazole-C12 (blue squares). Source data are provided as Supplementary Data file.

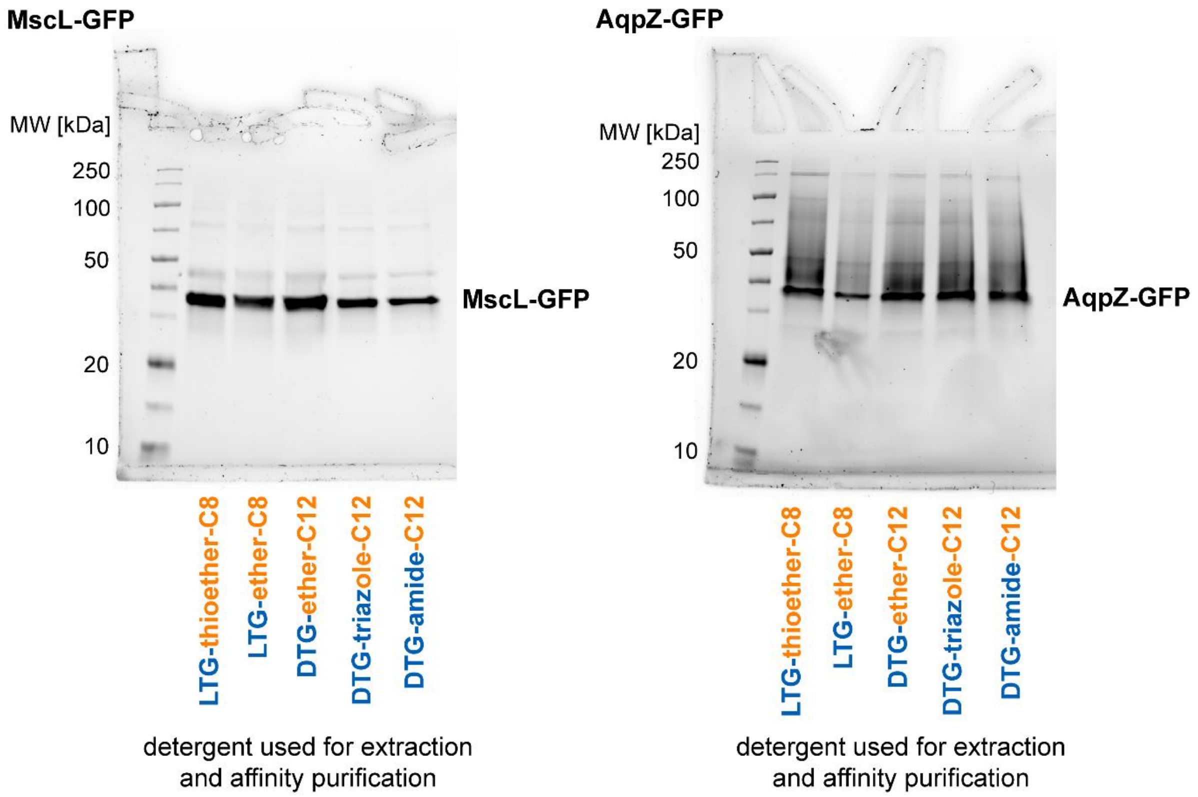

Supplementary Figure 6. SDS PAGE analysis of membrane protein preparations. Images of SDS PAGE gels were taken from membrane protein samples (MscL-GFP or AqpZ-GFP) that were purified with different detergents. SDS PAGE gels confirm that the relevant proteins were purified with comparable purity. Protein-detergent combinations for which no protein yields were detected by UV/VIS spectroscopy upon extraction and affinity purification were not analysed by SDS PAGE. Source data are provided as Supplementary Data file.

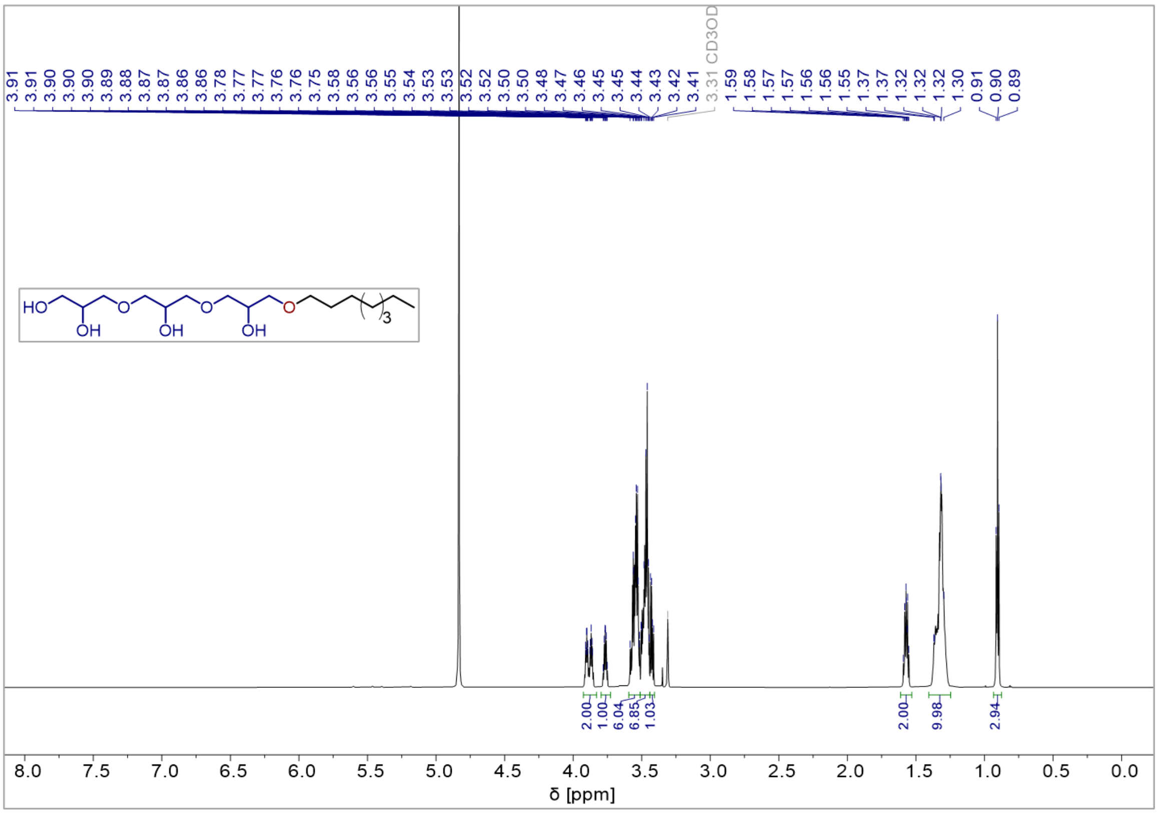

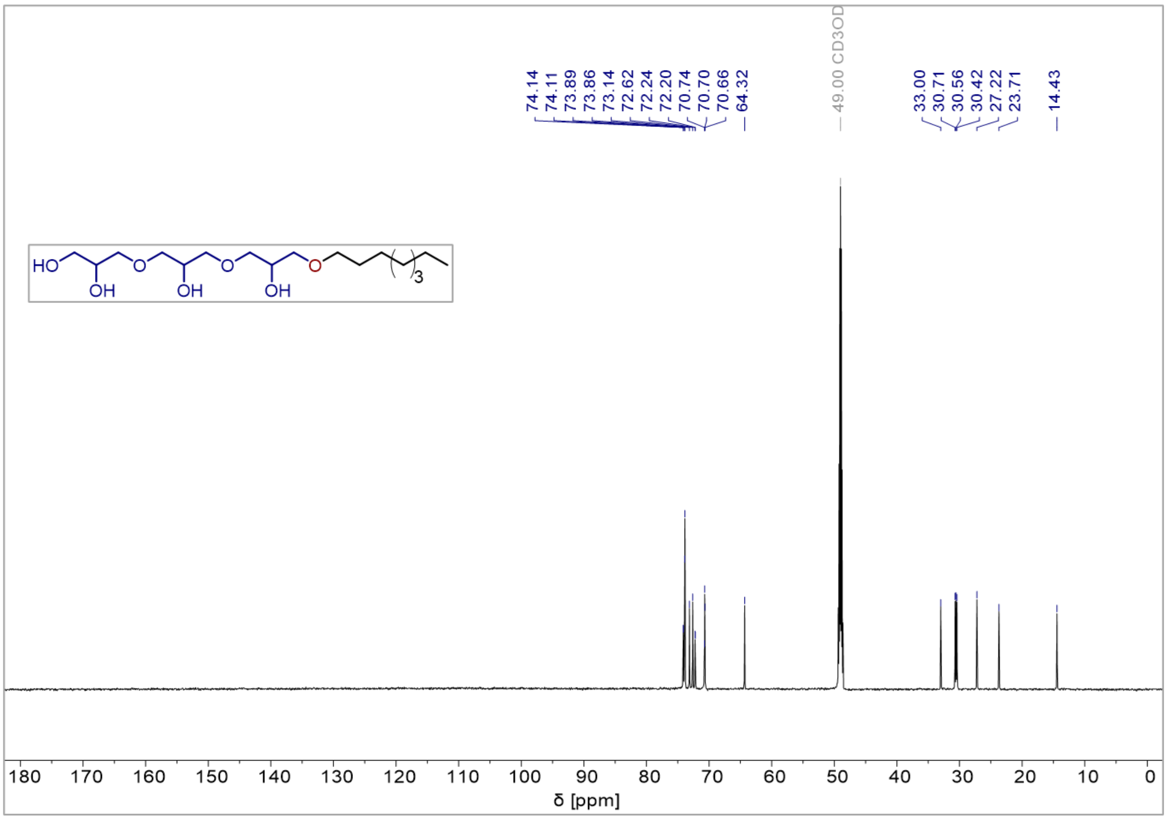

Supplementary Figure 7: **NMR data of LTG-ether-C8.** ^{1}H and ^{13}C NMR spectra of compound **11** (LTG-ether-C8).

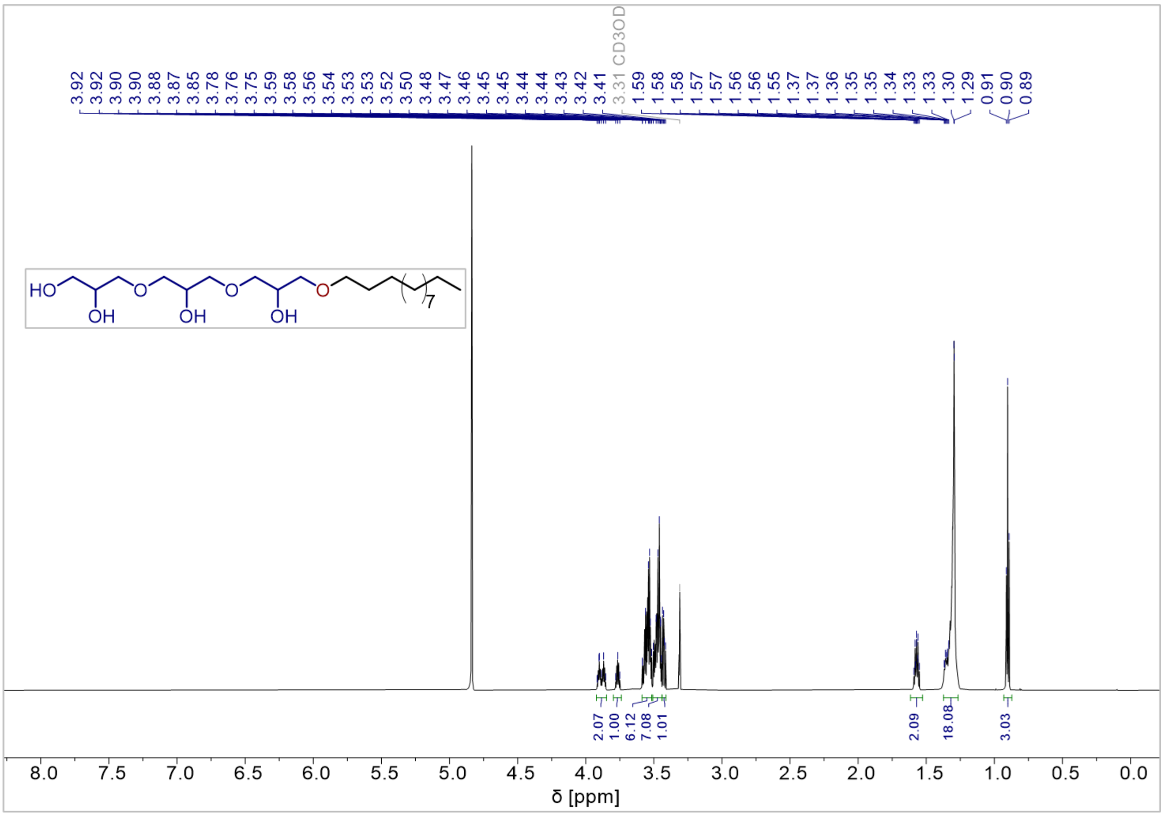

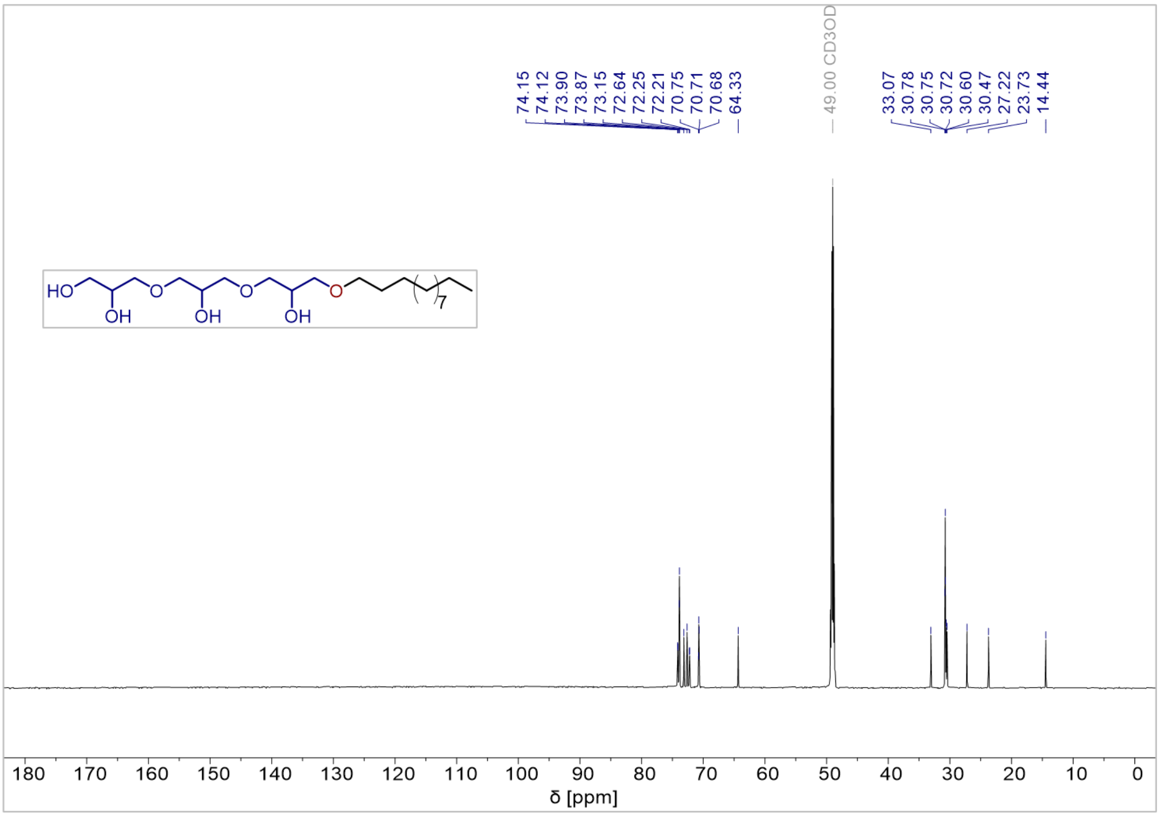

Supplementary Figure 8: **NMR data of LTG-ether-C12.** ^{1}H and ^{13}C NMR spectra of compound **12** (LTG-ether-C12).

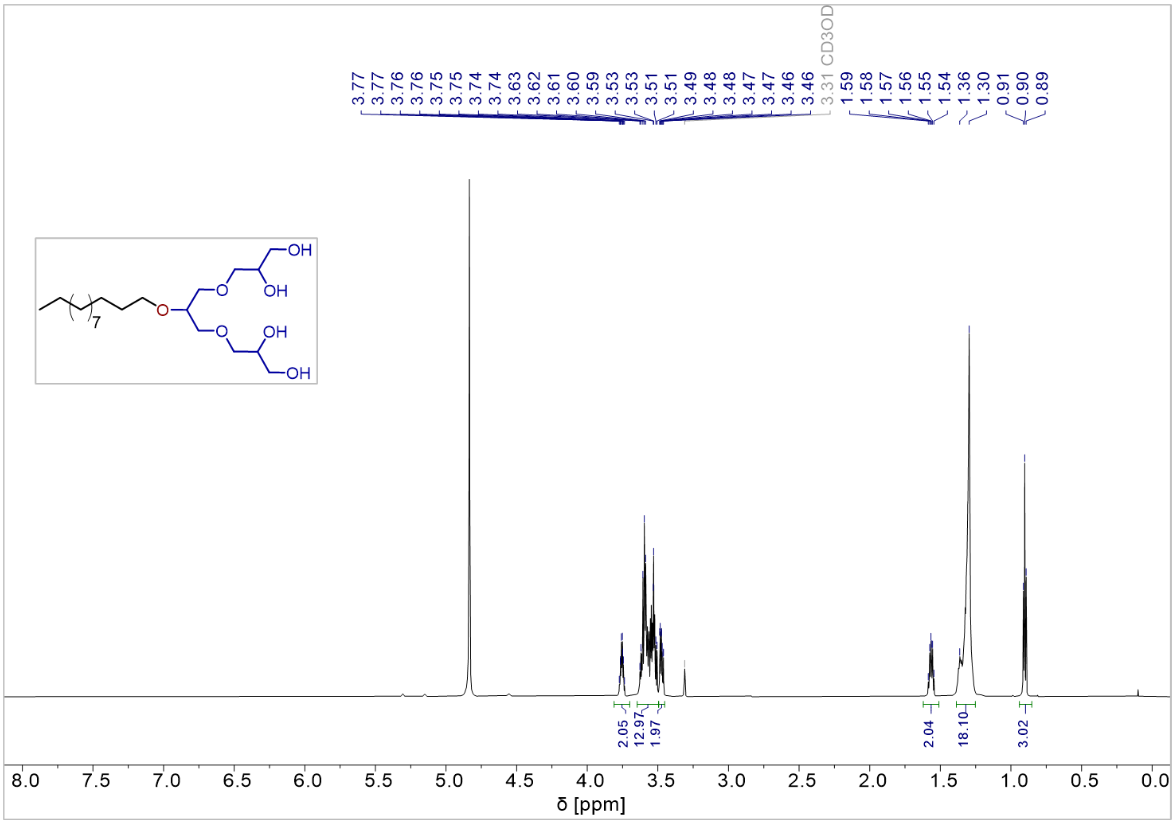

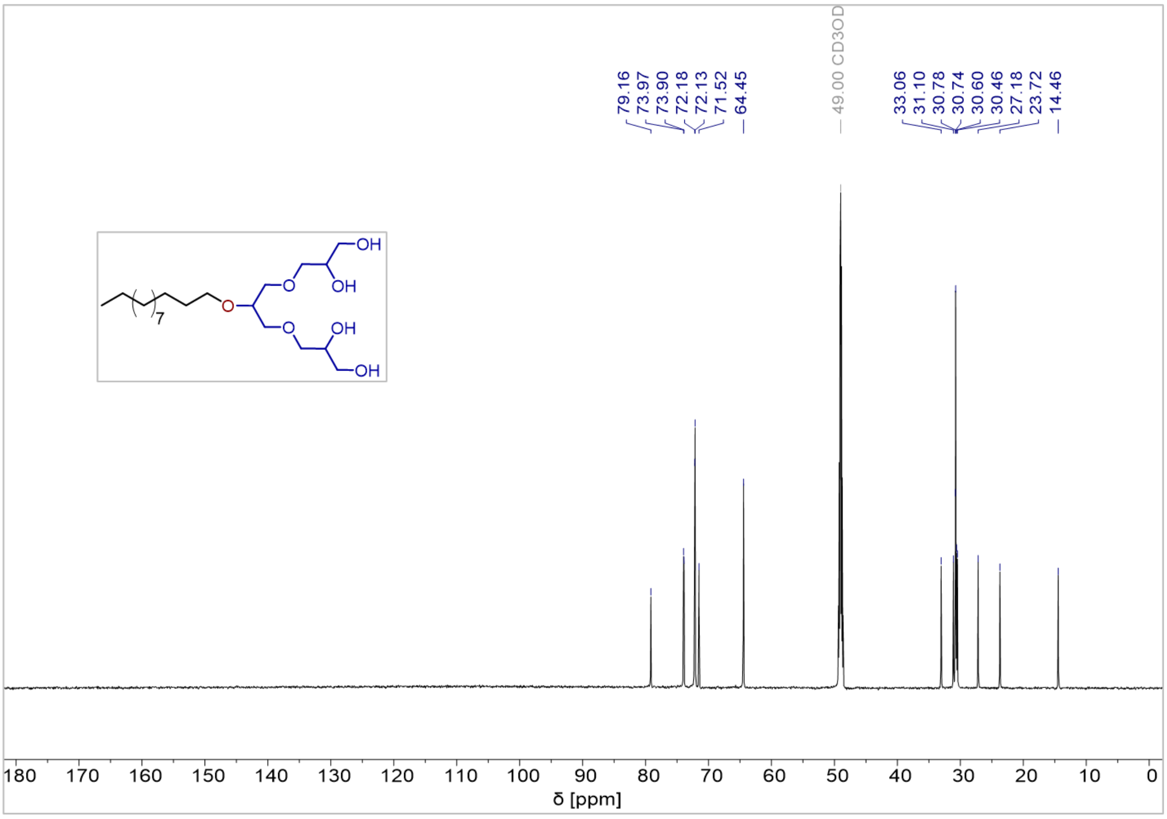

Supplementary Figure 9: **NMR data of DTG-ether-C12.** ^{1}H and ^{13}C NMR spectra of compound **13** (DTG-ether-C12).

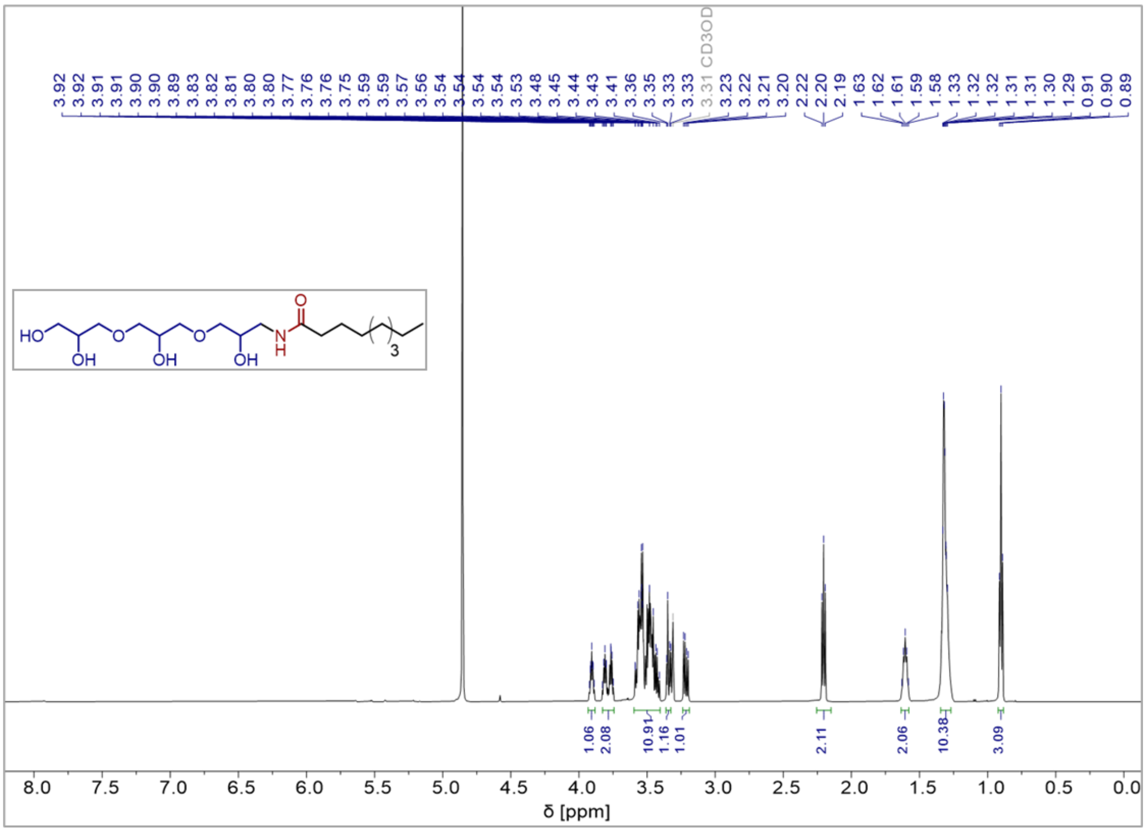

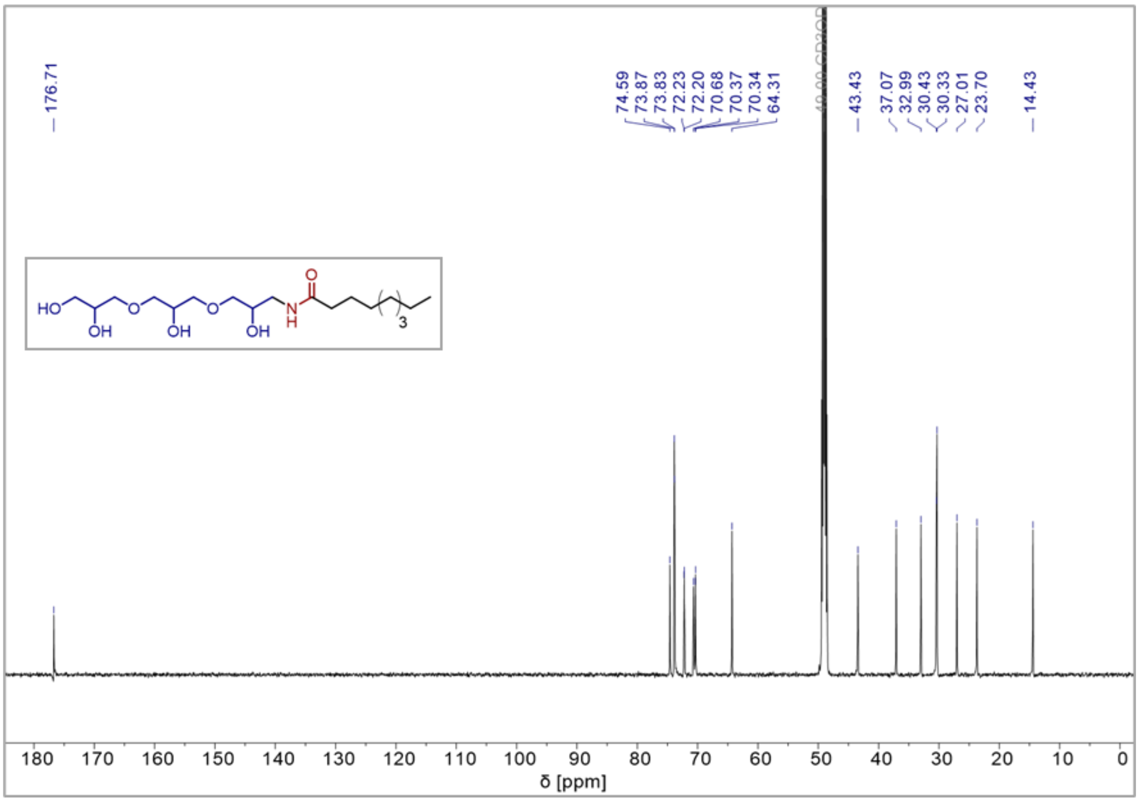

Supplementary Figure 10. NMR data of LTG-amide-C8. ^{1}H and ^{13}C NMR spectra of compound **14** (LTG-amide-C8).

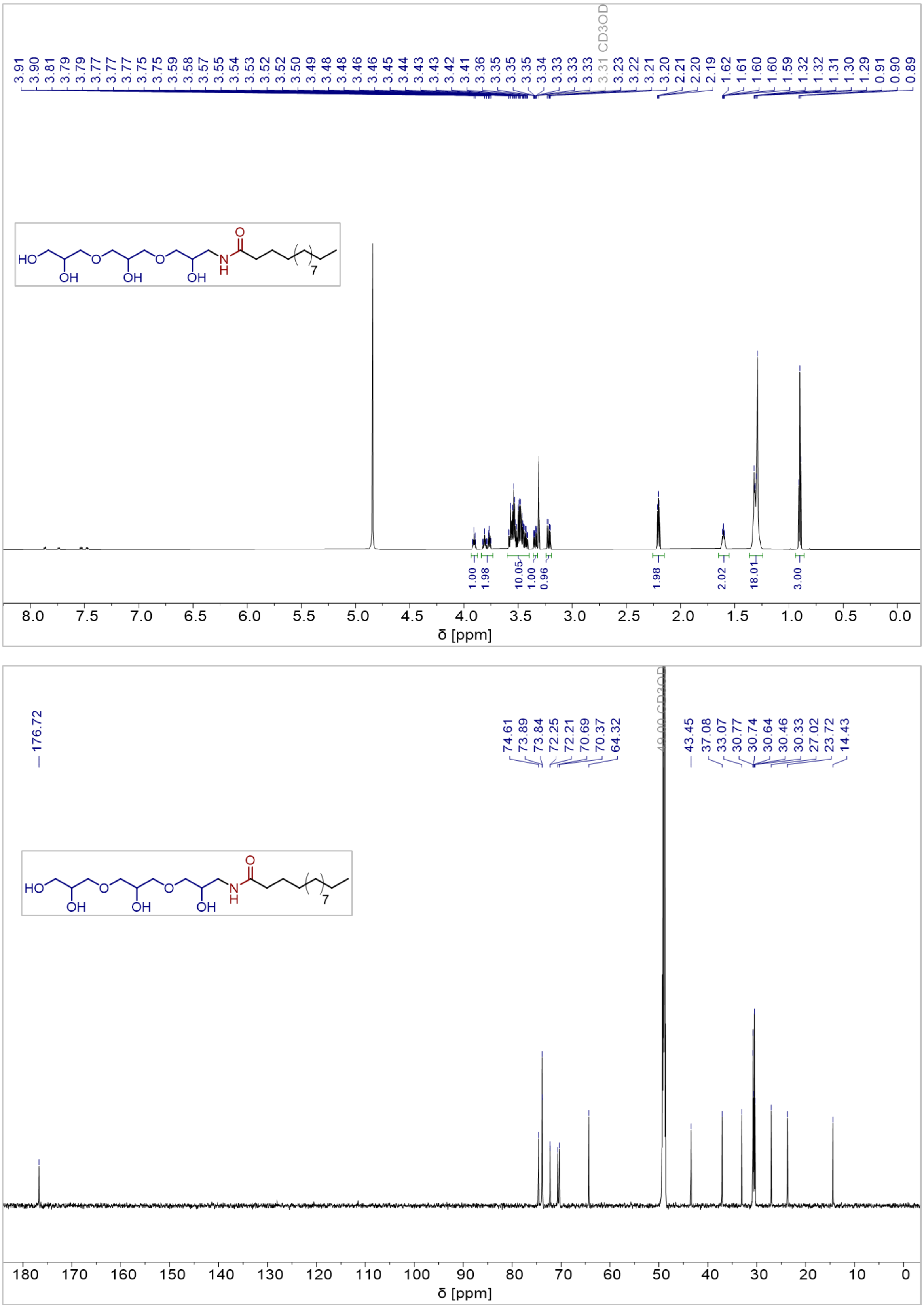

Supplementary Figure 11. NMR data of LTG-amide-C12. ^{1}H and ^{13}C NMR spectra of compound **15** (LTG-amide-C12).

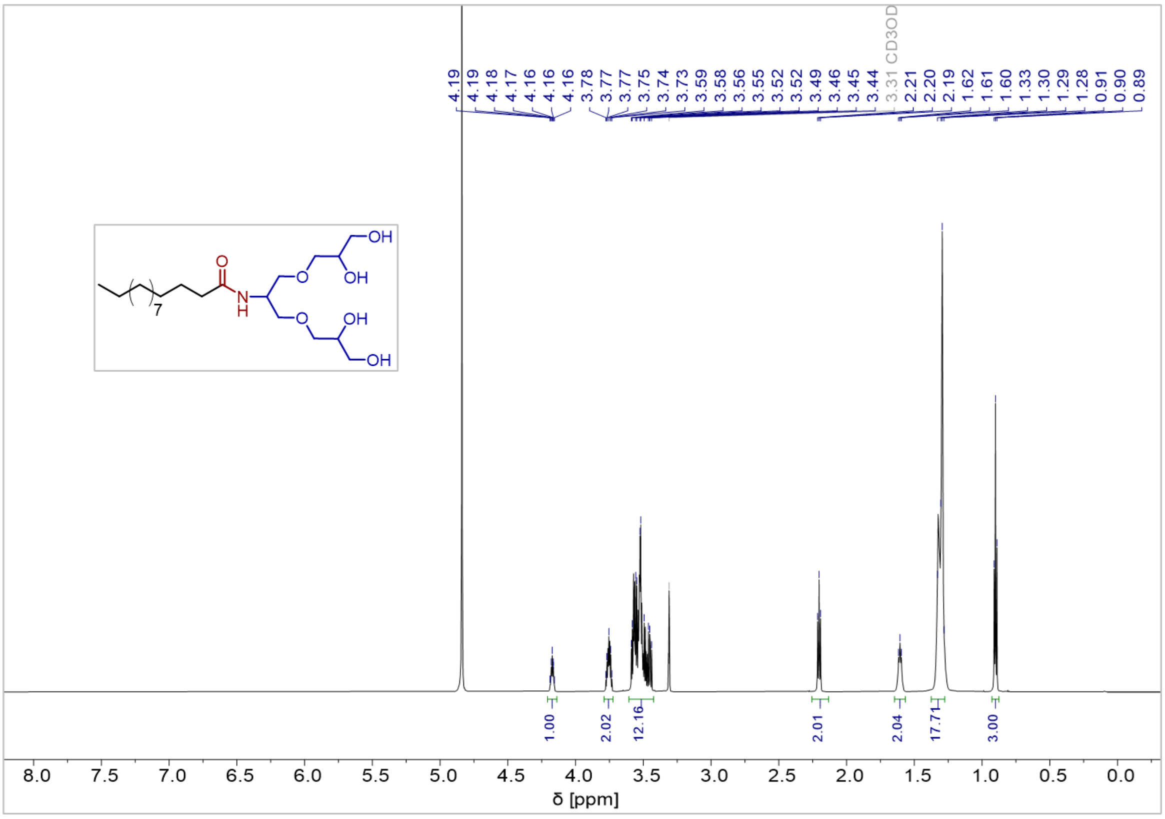

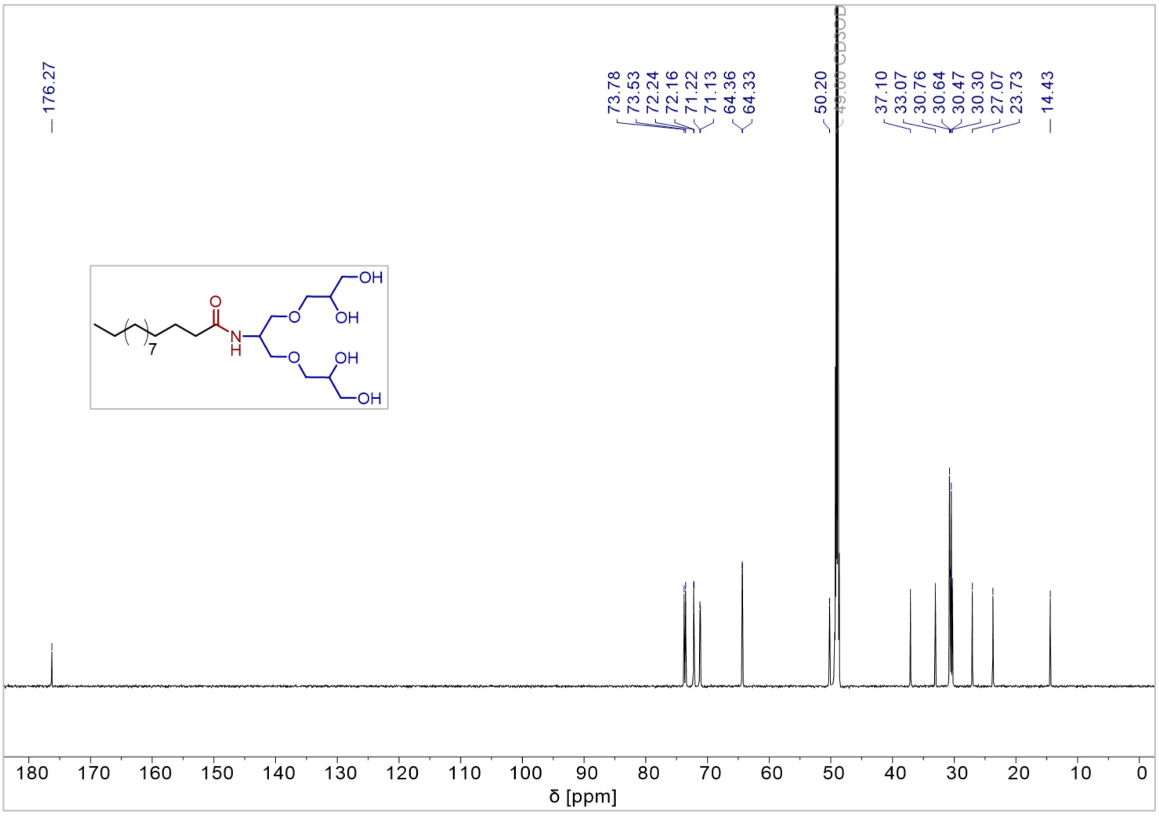

Supplementary Figure 12. NMR data of DTG-amide-C12. ^{1}H and ^{13}C NMR spectra of compound **16** (DTG-amide-C12).

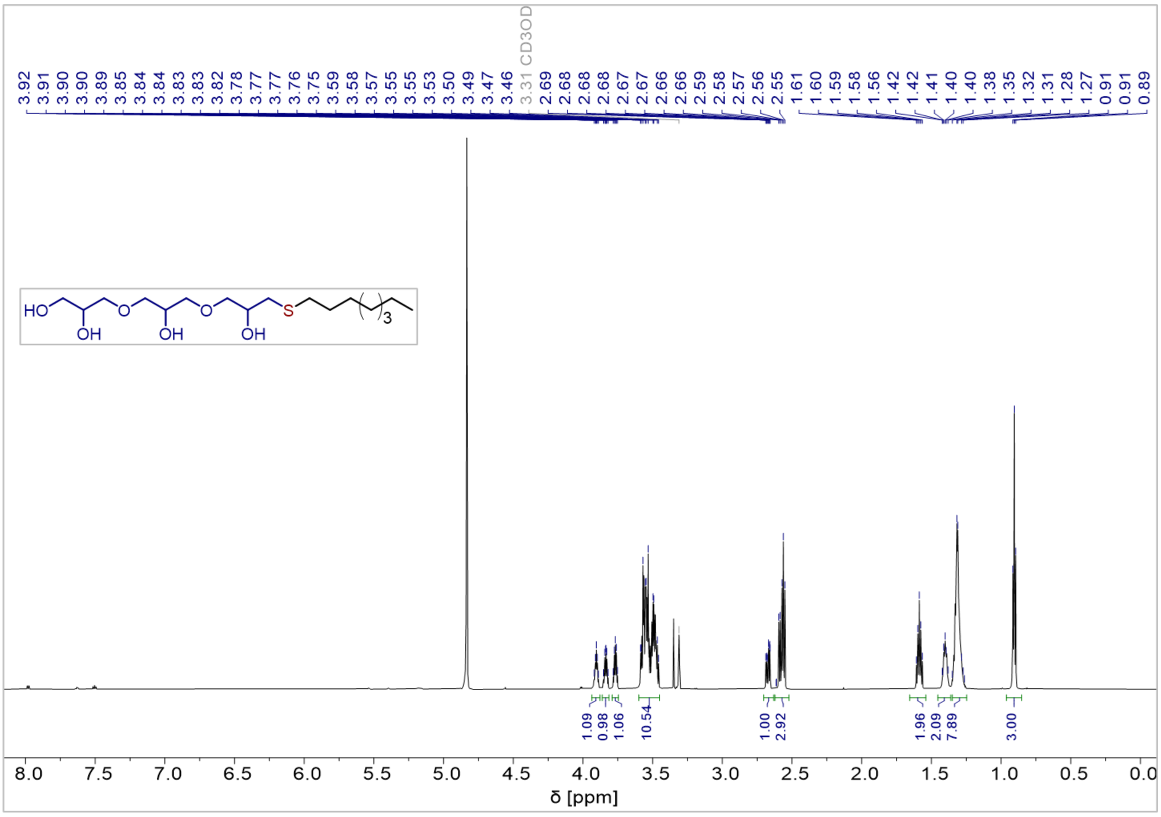

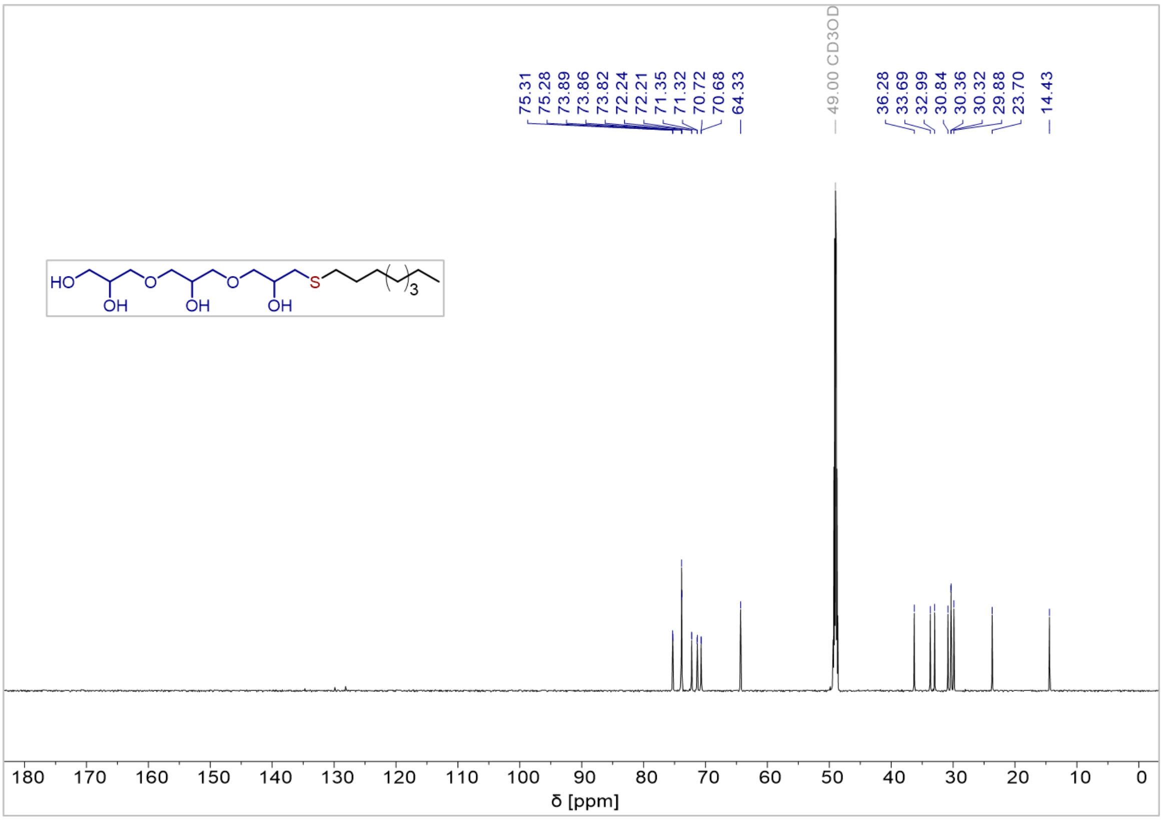

Supplementary Figure 13. NMR data of LTG-thioether-C8. ^{1}H and ^{13}C NMR spectra of compound **17** (LTG-thioether-C8).

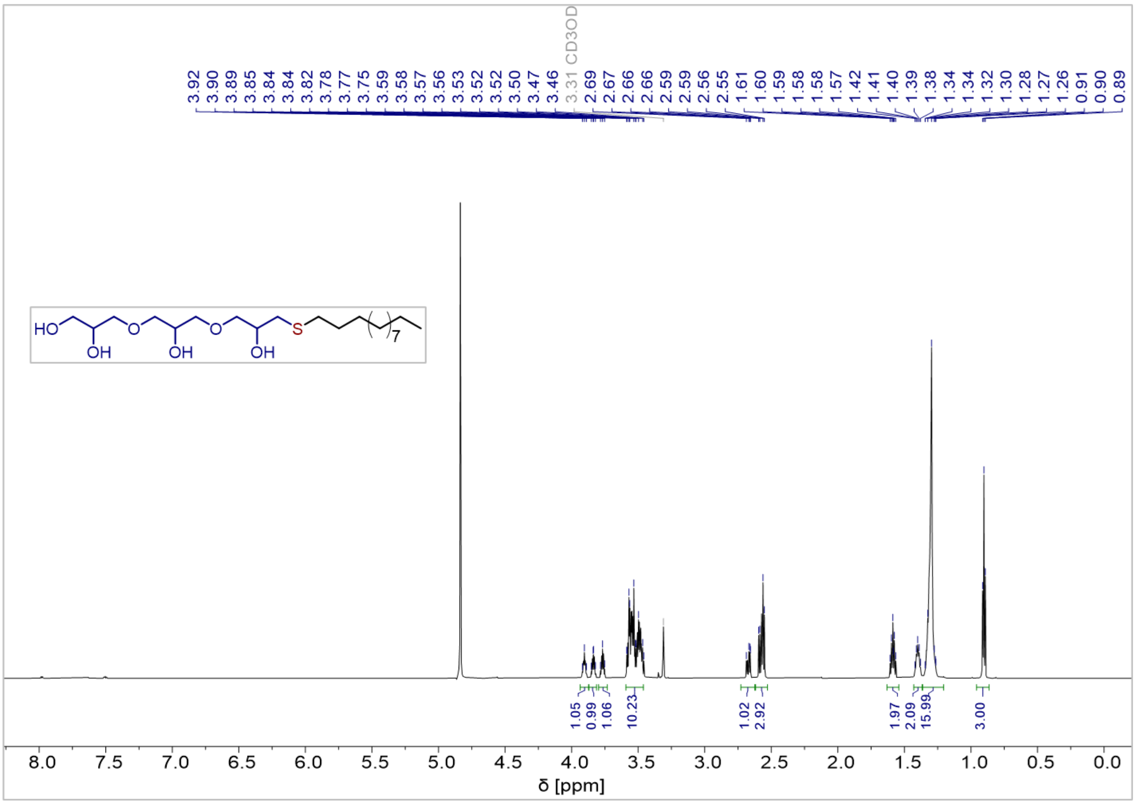

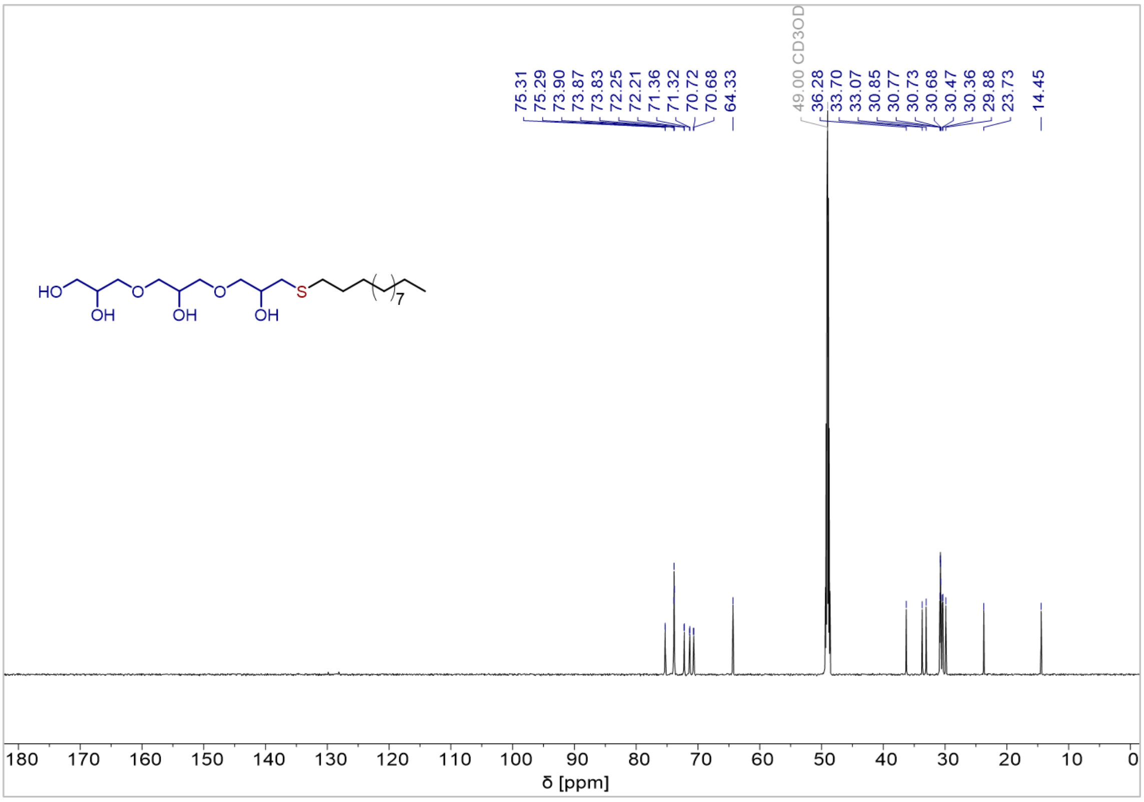

Supplementary Figure 14. NMR data of LTG-thioether-C12. ^{1}H and ^{13}C NMR spectra of compound **18** (LTG-thioether-C12).

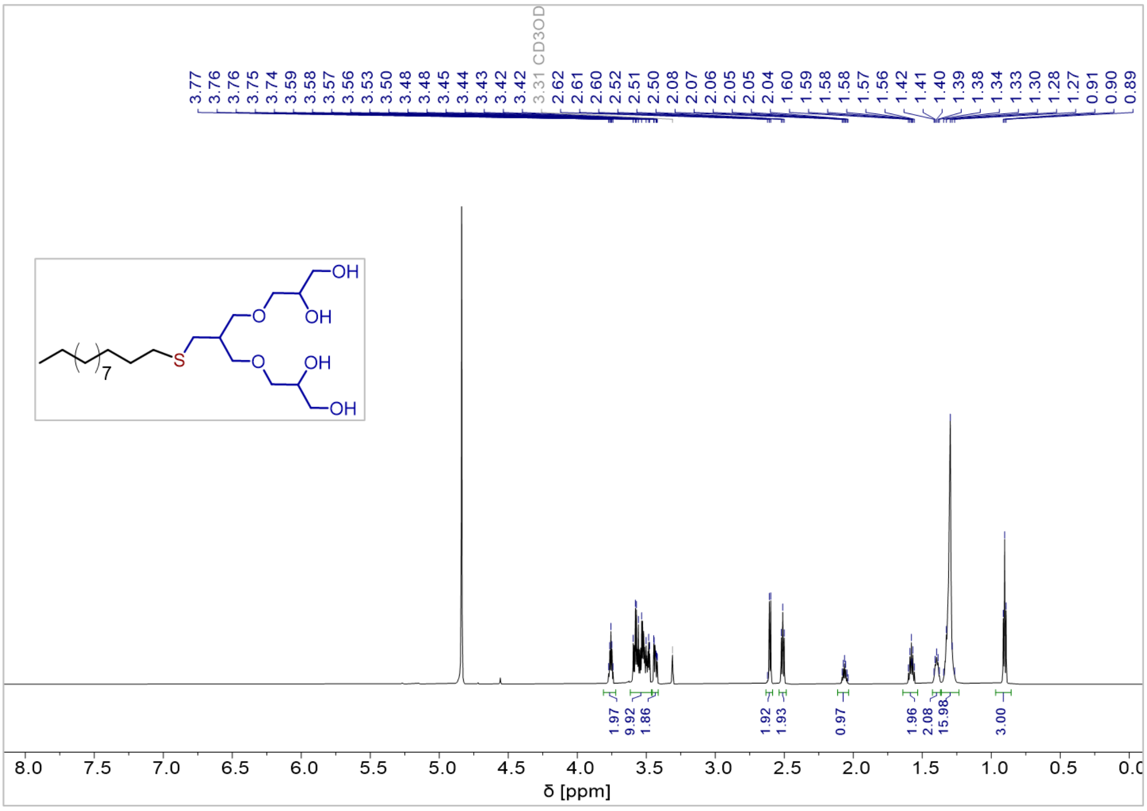

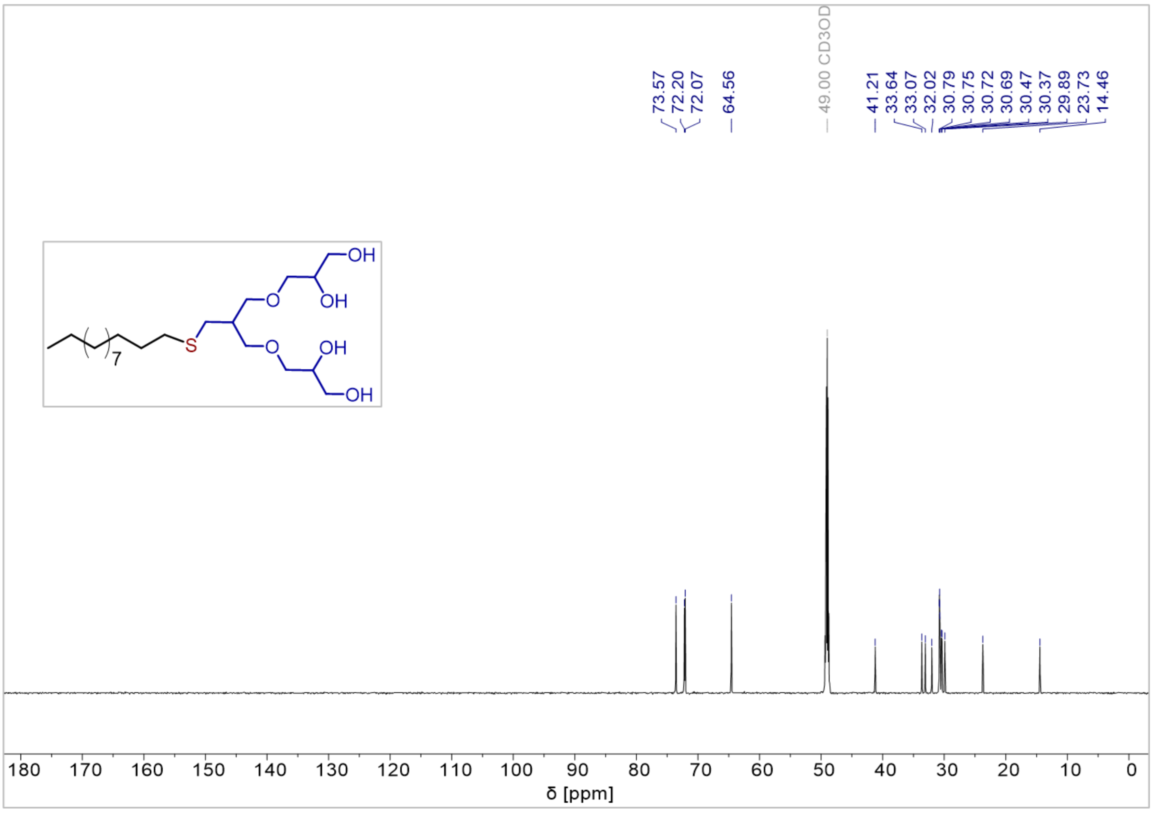

Supplementary Figure 15. NMR data of DTG-thioether-C12. ^{1}H and ^{13}C NMR spectra of compound **19** (DTG-thioether-C12).

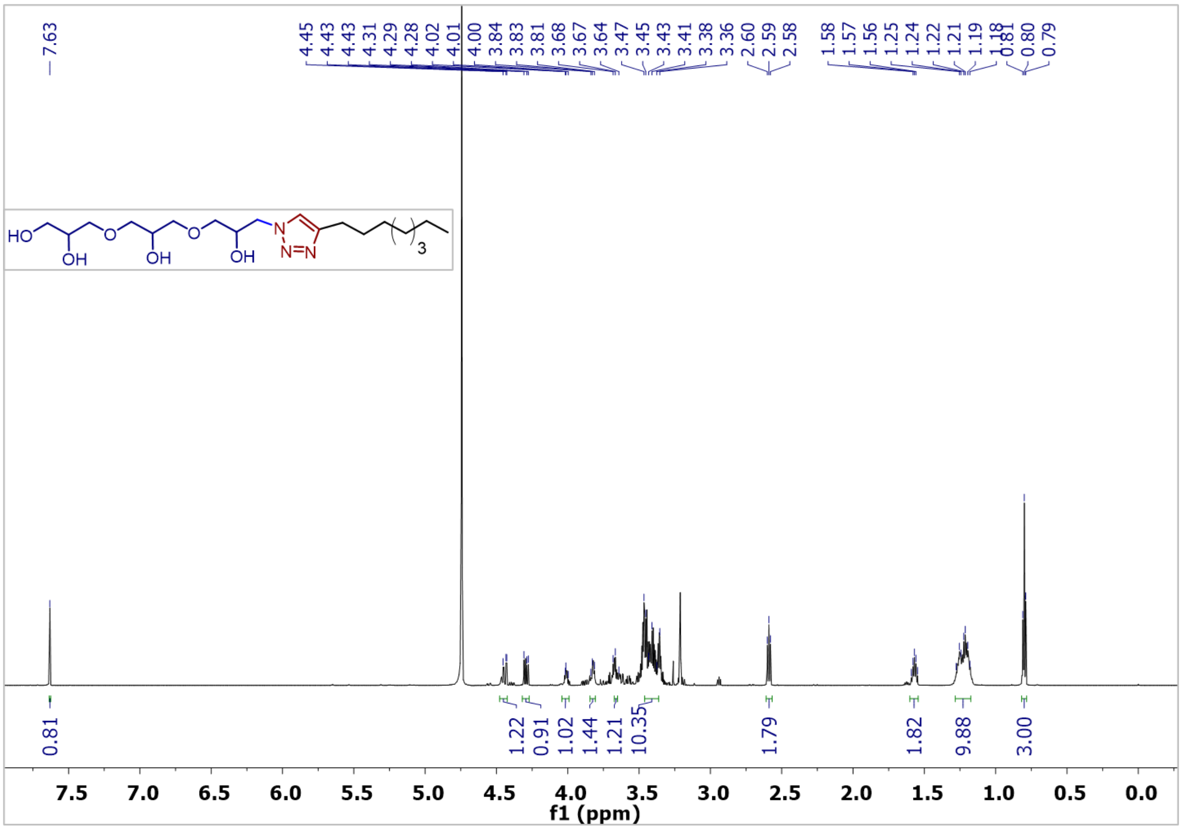

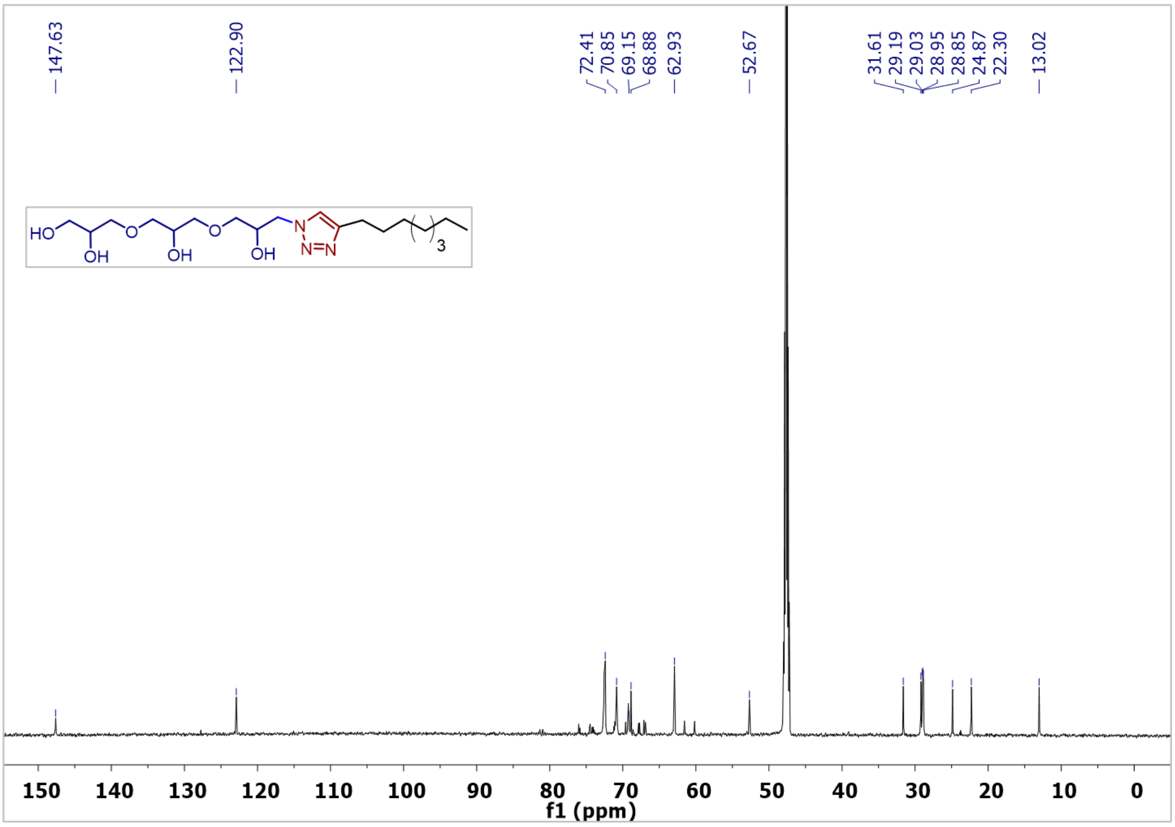

Supplementary Figure 16. NMR data of LTG-triazole-C8. ^{1}H and ^{13}C NMR spectra of compound **20** (LTG-triazole-C8).

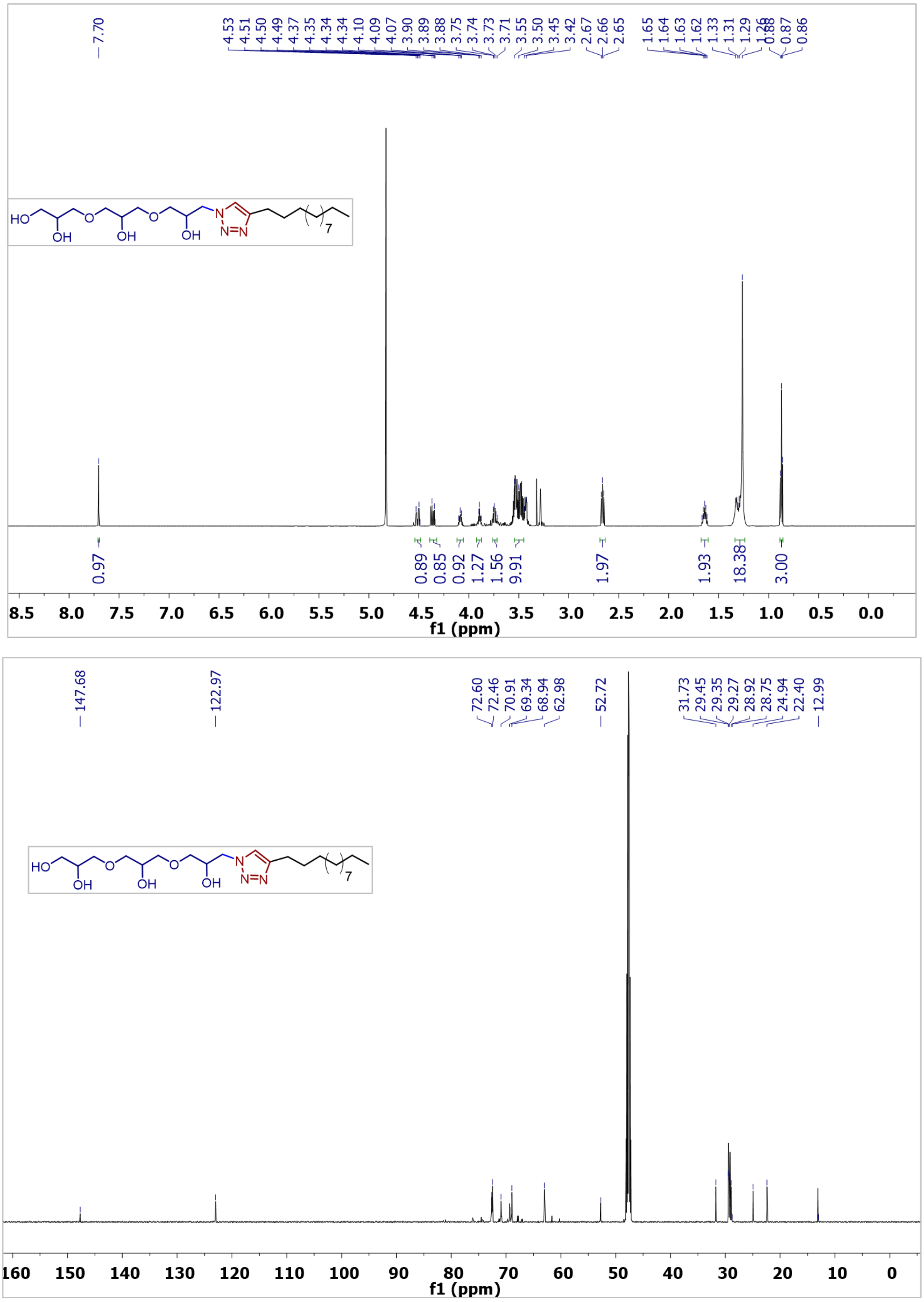

Supplementary Figure 17. NMR data of LTG-triazole-C12. ^{1}H and ^{13}C NMR spectra of compound **21** (LTG-triazole-C12).

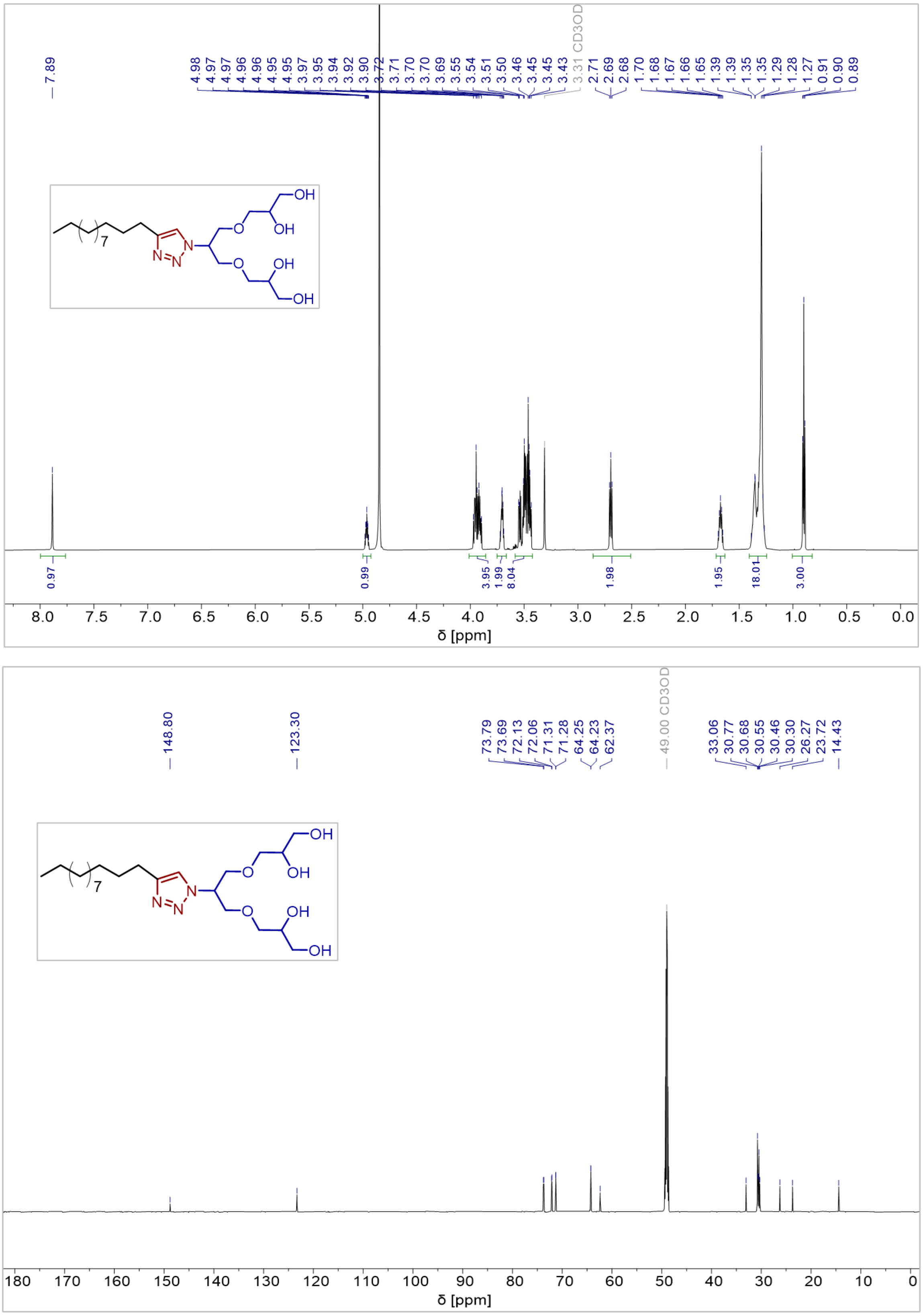

Supplementary Figure 18. NMR data of DTG-triazole-C12. ^{1}H and ^{13}C NMR spectra of compound **22** (DTG-triazole-C12).

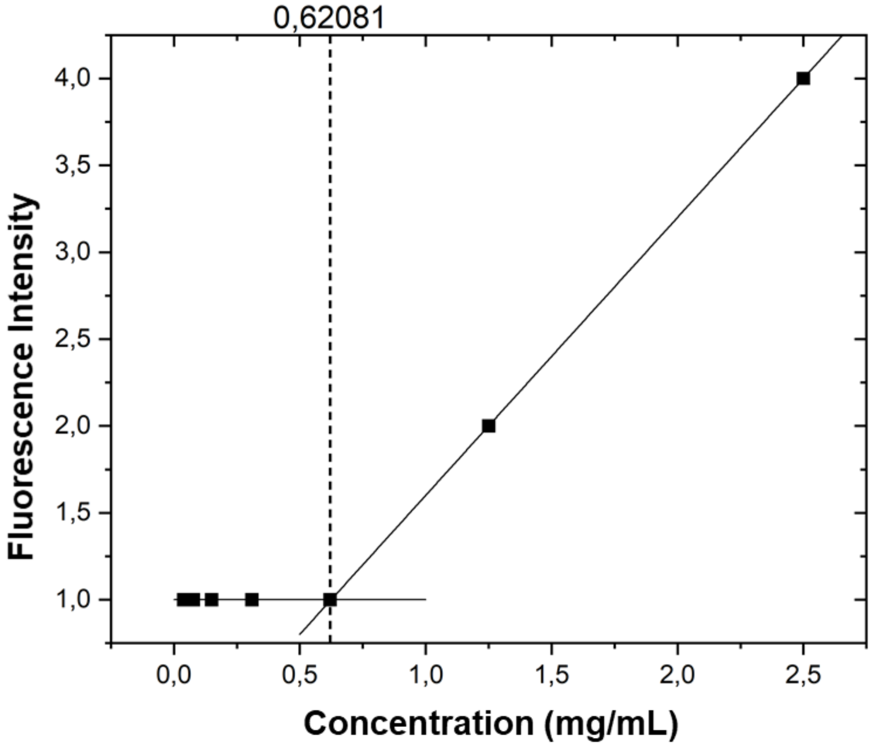

Supplementary Figure 19. Fluorescence-based critical micelle concentration analysis. Representative plot showing fluorescence intensities (black squares) obtained from solubilized Nile Red in water below and above the critical micelle concentration (cmc) of LTG-S-C8. The intersection between both regions was extrapolated and taken as cmc. Source data are provided as Supplementary Data file.

2. Supplementary Tables

Supplementary Table 1: Overview of aggregation parameters. Summary of parameters relevant for describing aggregation properties of non-ionic triglycerol detergents that are subject of this study, including water solubility, hydrophilic-lipophilic balance (HLB) values, packing parameter values (p), critical micelle concentration values (cmc), diffusion coefficient (D_{coeff}) values of particles formed in water above cmc, hydrodynamic radii (Dh) of particles formed in water above cmc, aggregate morphology confirmed by cryogenic electron microscopy (Cryo-EM).

detergent	water solubility [w% w/v]	HLB‡ [a.u.]	cmc [$mg{\cdot}mL^{-1}$]	D_{coeff} [$\mu m{\cdot}s^{-2}$]	D_h[nm]	aggregate morphology
LTG-thioether-C8	> 10	12.11	0.62	102	4.1	micelle
LTG-ether-C8	> 10	12.66	1.20	102	4.8	micelle
LTG-triazole-C8	> 10	13.14	1.20	102	4.8	micelle
LTG-amide-C8	> 10	14.03	0.99	8, 75, 158	0.6, 2.6, 5.6	micelle*
LTG-thioether-C12	< 2.5	11.27	n.t.	n.t.	n.t.	n.t.
LTG-ether-C12	< 2.5	11.71	n.t.	n.t.	n.t.	n.t.
LTG-triazole-C12	> 10	11.54	0.48	7	n.a.	worm-like micelle
LTG-amide-C12	< 2.5	12.23	n.t.	n.t.	n.t.	n.t.
DTG-thioether-C12	> 10	10.51	0.26	7	n.a.	worm-like micelle
DTG-ether-C12	> 10	10.93	0.28	65	5.6	micelle
DTG-triazole-C12	> 10	11.54	0.27	75	5.6	micelle
DTG-amide-C12	> 10	12.23	0.29	102	4.8	micelle
[G1]-ether-C14	< 5	-	0.19	22.8	n.a.	worm-like micelle
[G1]-thioether-CF6	< 5	-	0.12	7.81	n.a.	worm-like micelle
[G2]-carbamate-Chol	< 5	-	0.17	102	4.8	micelle
[G2]-triazole-DC12	<2.5	-	0.07	7	n.a.	worm-like micelle

‡ values were calculated from molecular detergent structures after assignment refinement of polar and non-polar building blocks guided by reversed phase HPLC as described in the manuscript

* – polydisperse particle size profile was obtained by DLS and D_{coeff} values of 75 - 158 $\mu m/s^2$ suggest weakly aggregating micelles, whose presence in solution could not be confirmed by Cryo-EM due to insufficient contrast

n.a. – not applicable, because Dh values are calculated with Stokes-Einstein equation based on the assumption that D_{coeff} values are obtained from spherical particles which could not be confirmed in this case by Cryo-EM

n.t. – not tested

Supplementary Table 2. Overview of HLB calculation parameters. Summary of parameters relevant for the calculation of HLB values, including name of the detergents [detergent], molecular weights of the detergents [MW], molecular weights of the detergent tails (without linker contributions) [MW_{tail}], HLB values (without linker contributions) [HLB], molecular weights of the detergent tails (with linker contributions) [$MW_{refinedtail}$], and refined HLB values (with linker contributions in tails) [$HLB_{refined}$].

[detergent]	[MW] / Da	[MW_{tail}] / Da	[HLB]	[$MW_{refinedtail}$] / Da	[$HLB_{refined}$] / Da
LTG-thioether-C8	368.53	145.28	12.11	145.28	12.11
LTG-ether-C8	352.47	129.22	12.66	129.22	12.66
LTG-triazole-C8	403.52	152.26	12.45	138.25	13.14
LTG-amide-C8	379.49	141.23	12.55	113.22	14.03
LTG-thioether-C12	424.64	187.37	11.17	185.33	11.27
LTG-ether-C12	408.58	185.33	10.92	169.34	11.71
LTG-triazole-C12	459.63	208.37	10.93	194.36	11.54
LTG-amide-C12	435.6	197.34	10.93	169.34	12.22
DTG-thioether-C12	424.64	187.37	11.17	201.39	10.51
DTG-ether-C12	408.58	185.33	10.92	185.33	10.92
DTG-triazole-C12	459.63	208.37	10.93	194.36	11.54
DTG-amide-C12	435.60	197.34	10.93	169.34	12.22

3. Detergent Synthesis

Synthesis of head groups

The head group synthesis of both dendritic and linear started from commercially available Glycidyl allyl ether (GAE). The synthetic details have been mentioned in **Supplementary Scheme 1 and 2**.

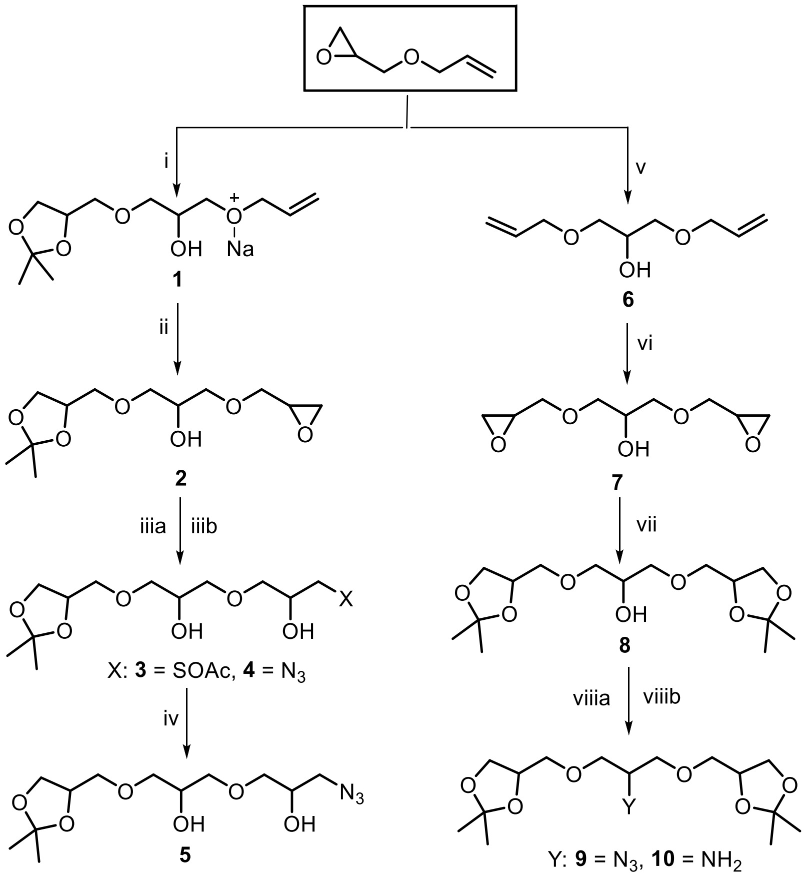

Supplementary Scheme 1: Synthesis of linear and dendritic head groups: i) Solketal, TBAB, NaOH, Toluene, 24 h, 60 °C; ii) mCPBA, DCM, rt, 24h; iiia) Thioacetic acid, H_2O, 12 h, rt.; iiib). NaN_3, Water, 50 °C, 24 h; iv) Pd/C, H_2, MeOH, 24 h; v) Ally alcohol, KOH, TBAB, Toluene, 60°C, 24h; vi) mCPBA, DCM, 24 h; vii) 1. Water, acetic acid, 100 °C 24 h, 2. 2,2-dimethoxy propane, ptsa, 24 h, rt. viiia) MsCl, TEA, NaN_3, DMF; viiib) Pd/C, H_2, MeOH, 24 h.

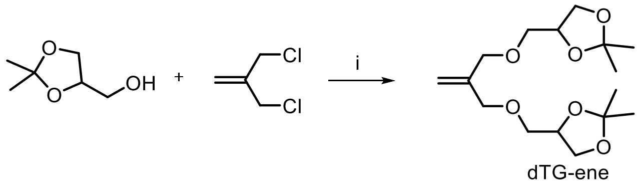

Supplementary Scheme 2: Synthesis of dTG-ene. i) NaH, KI, 15-crown-5, 18-crown-6, THF, 65 °C, 24 h.

Synthesis of compound 1

GAE (10 g, 1 eq) and solketal (13.8 g, 1.2 eq) were weighed in the round bottom flask. Both reactants were dissolved in the required amount of Toluene (65 mL). In the same flask, KOH (5.4 g, 1.1 eq) was added followed by the addition of TBAI (2.8 g, 0.1 eq). The whole reaction mixture was stirred for 30 minutes in an ice bath then the flask was heated at 50 ^{o}C for 24 hours. The progress of the reaction mixture was monitored by TLC using hexane and ethyl-acetate as an eluent. After the completion of the reaction, Toluene was removed under reduced pressure and then obtained residue was extracted with water and ethyl acetate (30 mL x 3). The organic layer was dried over sodium sulphate and concentrated under reduced pressure. The desired product was obtained after column purification in 65% yield.

^{1}**H NMR** (400 MHz, METHANOL-D_3) δ 5.97 – 5.87 (m, 1H), 5.37 – 5.08 (m, 2H), 4.29 – 4.23 (m, 1H), 4.18 – 4.01 (m, 3H), 3.89 – 3.84 (m, 1H), 3.76 – 3.71 (m, 1H), 3.59 – 3.42 (m, 6H), 1.38 (s, 3H), 1.33 (s, 3H) ppm; ^{13}**C NMR** (101 MHz, METHANOL-D_3) δ 136.10, 117.16, 110.43, 77.70, 76.09, 74.06, 73.37, 73.23, 72.53, 70.58, 67.53, 63.83, 27.06, 25.65 ppm; **MS (ESI)** m/z = 269.1359 $[M+Na]^+$ calculated: $C_{12}H_{22}NaO_5^+$: 269.1363

Synthesis of compound 2

Compound **1** (5 g, 1 eq) was dissolved in DCM (20 mL) and stirred in an ice bath to maintain the temperature at 0 ^{o}C. In a separate flask the required amount of mCPBA (5.9 g, 1.7 eq) was dissolved in DCM (30 mL) and then added to the reaction flask while maintaining the ice bath condition. After the complete addition, the whole reaction mixture was stirred at room temperature for 48 h. The progress of the reaction mixture was monitored by TLC using methanol and DCM as an eluent. After the completion of the reaction, the reaction mixture was washed with sodium thiosulphate (30 ml x 2) followed by washing with Sodium bicarbonate solution (30 mL x 3), the organic layers were collected, dried over sodium sulphate, and concentrated under reduced pressure. The desired product was obtained after column purification in a 62% yield.

^{1}**H NMR** (400 MHz, METHANOL-D_3) δ 4.30 – 4.24 (m, 1H), 4.07 – 4.03 (m 1H), 3.88 – 3.79 (m, 2H), 3.76 – 3.70 (m, 1H), 3.60 – 3.48 (m, 6H), 3.38 – 3.33 (m, 1H), 3.11 – 3.13 (m, 1H), 2.82 – 2.77 (t, J = 4.6 Hz, 1H), 2.62 – 2.60 (m, 1H), 1.39 (s, 3H), 1.33 (s, 3H) ppm; ^{13}**C NMR** (101 MHz, METHANOL-D_3) δ 110.48, 79.83, 73.98, 73.92, 73.79, 73.72, 73.41, 73.26, 70.60, 67.55, 51.86, 44.60, 27.04, 25.64 ppm; **MS (ESI)** m/z = 263.1489; $[M+H]^+$ (Calculated: $C_{12}H_{23}O_6^+$: 263.1143).

Synthesis of compound 3

Compound 2 (2 g, 1 eq) was dissolved in water (15 mL), followed by the addition of thioacetic acid (1.2 eq). The reaction mixture was stirred at room temperature for 24 h. The progress of the reaction mixture was monitored by TLC using methanol and DCM. After the completion of the reaction, the reaction mixture was washed with sodium bicarbonate solution (30 mL x 3) and DCM (3 x30 mL), the organic layer was collected and dried over sodium sulphate and concentrated under reduced pressure. The desired product was obtained after column purification with a 55 % yield.

^{1}H NMR (500 MHz, MeOD) δ 4.34 – 4.20 (m, 1H), 4.05 (dd, *J* = 8.3, 6.5 Hz, 1H), 3.90 – 3.86 (m, 1H), 3.83 – 3.77 (m, 1H), 3.74 – 3.72 (m, 1H), 3.58 – 3.44 (m, 8H), 3.16 – 3.10 (m, 1H), 2.97 (dd, *J* = 13.7, 6.9 Hz, 1H), 2.33 (s, 3H), 1.39 (s, 3H), 1.33 (s, 3H) ppm; **^{13}C NMR** (151 MHz, MeOD): δ 172.28, 110.52, 79.22, 76.15, 73.93, 73.46, 72.30, 71.28, 70.59, 67.59, 27.07, 25.65, 20.78 ppm; **MS (ESI)** m/z = 361.1291; $[M+Na]^+$ (calculated:: $C_{14}H_{26}NaO_7S^+$: 361.1143).

Synthesis of compound 4

The synthesis of compound 4 was achieved by dissolving compound 2 (1.5 g, 1 eq) in water followed by the addition of sodium azide (0.7 g, 2 eq). The whole reaction mixture was stirred at 50 °C for 24 h. After the completion of the reaction, the reaction mixture was extracted with DCM (30 mL x 3), and the organic layer was dried over sodium sulphate and concentrated under reduced pressure. The desired product was obtained after column purification with a 67 % yield.

^{1}H NMR (500 MHz, METHANOL-*D*$_3$) δ 4.31 – 4.24 (m, 1H), 4.08 – 4.05 (m, 1H), 3.91 – 3.87 (m, 2H), 3.75 – 3.72 (m, 1H), 3.58 – 3.48 (m, 8H), 3.39 – 3.36 (m, 1H), 3.32 – 3.29 (m, 1H), 1.40 (s, 3H), 1.34 (s, 3H) ppm; **^{13}C NMR** (126 MHz, METHANOL-*D*$_3$) δ 110.52, 76.17, 73.95, 73.87, 73.45, 70.82, 70.61, 67.56, 54.84, 27.04, 25.63 ppm; **MS (ESI)** m/z = 328.1491; $[M+Na]^+$ (calculated: $C_{12}H_{23}N_3NaO_6^+$: 328.1479).

Synthesis of compound 5

The amino derivative was synthesized by the reduction of compound **4** where compound 4 (1g) was dissolved in methanol, and further Pd/C (10%) was added to the flask. The whole reaction was stirred under a hydrogen atmosphere (5 bar) for 24 h. After the completion of the reaction, the Pd/C was filtered, and the methanol was concentrated to get the desired compound 5 in 95 % yield.

^{1}H NMR (400 MHz, METHANOL-*D*$_3$) δ 4.30 – 4.25 (m, 1H), 4.08 – 4.04 (m, 1H), 3.94 – 3.86 (m, 1H), 3.81 – 3.71 (m, 2H), 3.58 – 3.49 (m, 2H), 2.94 – 2.61 (m, 2H), 1.39 (s, 3H), 1.33 (s, 3H) ppm; **^{13}C NMR** (101 MHz, METHANOL-*D*$_3$) δ 110.54, 76.12, 75.32, 73.83, 73.39, 70.58,

69.66, 67.47, 27.02, 25.60, 21.98 ppm; **MS (ESI)** m/z = 280.1776; $[M+H]^+$ (calculated: $C_{12}H_{26}NO_6^+$: 280.1775).

Synthesis of compound 6

GAE (10 g, 1 eq) was dissolved in the required amount of toluene (40 mL), followed by the addition of allyl alcohol (14.2 g, 5 eq). Further, KOH (9.2 g, 2 eq) and TBAB (2.82 g, 0.1eq) were added to the reaction flask. The combined reaction flask was stirred in an ice bath for 30 minutes. Later the reaction flask has been heated up at 50 °C and left over for 24 h. The progress of the reaction mixture was monitored by TLC. After the completion of the reaction, toluene was removed under reduced pressure and then obtained residue was extracted with water and ethyl acetate (30 mL x 3). The organic layer was dried over sodium sulphate and concentrated under reduced pressure. The desired product was obtained after column purification with 87 % yield.

^{1}H NMR (600 MHz, CHLOROFORM-*D*) δ 5.93 – 5.84 (m, 2H), 5.25 (dd, *J* = 17.3, 1.7 Hz, 2H), 5.17 (dd, *J* = 10.4, 1.5 Hz, 2H), 4.00 (dt, *J* = 5.7, 1.5 Hz, 4H), 3.98 – 3.93 (m, 1H), 3.51 (dd, *J* = 9.8, 4.4 Hz, 2H), 3.45 (dd, *J* = 9.7, 6.3 Hz, 2H), 2.60 (d, *J* = 4.3 Hz, 1H) ppm; **^{13}C NMR** (151 MHz, CHLOROFORM-*D*) δ 134.60, 117.33, 72.43, 71.39, 69.61 ppm; **MS (ESI)** m/z = 179.0964; $[M+Na]^+$ (calculated for $C_9H_{16}NaO_3^+$: 179.0992).

Synthesis of compound 7

Compound **6** (6 g, 1 eq) was dissolved in DCM (45 mL) and stirred in an ice bath to maintain the temperature at 0 °C. In a separate flask the required amount of mCPBA (21.6 g, 3.5 eq) was dissolved in DCM (35 mL) and then added to the reaction flask while maintaining the ice bath condition. After the complete addition, the whole reaction mixture was stirred at room temperature for 48 h. The progress of the reaction mixture was monitored by TLC. After the completion of the reaction, the reaction mixture was washed with sodium thiosulphate (30 ml x 2) to quench the reaction followed by washing with Sodium bicarbonate solution (30 mL x 3), the organic layer was dried over sodium sulphate and concentrated under reduced pressure. The desired product was obtained after column purification in a 68 % yield.

^{1}H NMR (600 MHz, METHANOL-*D*$_4$) δ 3.92 – 3.86 (m, 1H), 3.82 – 3.75 (m, 2H), 3.60 – 3.45 (m, 8H), 3.38 – 3.34 (m, 1H), 3.16 – 3.14 (m, 1H), 2.78 – 2.77 (m, 1H), 2.62 – 2.61 (m, 1H) ppm; **^{13}C NMR** (151 MHz, METHANOL-*D*$_4$) δ 73.84, 73.23, 72.21, 70.64, 64.31, 51.89, 44.64 ppm; **MS (ESI)** m/z = 227.0890; $[M+Na]^+$ (calculated: $C_9H_{16}NaO_5^+$: 227.0810).

Synthesis of compound 8

Compound **7** (4 g, 1 eq) was hydrolysed with water (15 mL) and a small amount of acetic acid (0.2 ml) which refluxed at 80 °C for 24 h. After the reaction completion, the water was removed and dried on rota vapor. Further in situ, 2,2 dimethoxy propane (10 eq) and PTSA (0.1 eq) were added to the crude obtained after the hydrolysis. The progress of the reaction mixture was monitored by TLC. After the completion of the reaction, the reaction mixture was neutralized with triethylamine (0.1 eq). The solvent was removed under reduced pressure, the obtained residue was washed with water and ethyl acetate (30 mL x 3). The organic layer was dried over sodium sulphate and concentrated under reduced pressure. The desired product was obtained after column purification in a 72 % yield.

^{1}H NMR (500 MHz, MeOD) δ 4.26 (p, *J* = 5.7 Hz, 2H), 4.06 – 4.04 (m, 2H), 3.87 – 3.85 (m, 1H), 3.74 – 3.72 (m, 2H), 3.55 – 3.50 (m, 8H), 1.39 (s, 6H), 1.33 (s, 6H) ppm; **^{13}C NMR** (151 MHz, MeOD) δ 109.17, 74.84, 72.70, 72.65, 72.14, 69.29, 66.29, 25.80, 24.40 ppm; **MS (ESI)** m/z = 343.1727+; $[M+Na]^+$ (calculated: $C_{15}H_{28}NaO_7^+$: 343.1780).

Synthesis of compound 9

Compound **8** (2.5 g, 1 eq) was dissolved in DCM (20 mL) and stirred in ice bath. TEA (2.3 g, 3 eq) and methane sulfonyl chloride (1.3 g, 1.5 eq) was added to the reaction flask under argon condition. After, 3 h DCM was removed and then DMF (20 mL) was added followed by the addition of NaN_3 (1.5 g, 3 eq). The whole reaction mixture was stirred for 24 h at 80 °C. The progress of the reaction mixture was monitored by TLC. After the completion of the reaction, DMF was removed under rota vapor. and the obtained crude was extracted with DCM (30 mL x 3). The organic layer was dried over sodium sulphate and concentrated under reduced pressure. The desired product was obtained after column purification with a 75 % yield.

^{1}H NMR (700 MHz, MeOD) δ 4.25 (tt, *J* = 6.4, 5.3 Hz, 2H), 4.05 (m, 2H), 3.77 – 3.69 (m, 3H), 3.68 – 3.63 (m, 2H), 3.61 – 3.51 (m, 6H), 1.39 (s, 6H), 1.33 (s, 6H) ppm; **^{13}C NMR** (176 MHz, MeOD) δ 110.52, 76.10, 76.08, 73.21, 73.16, 72.17, 72.07, 67.46, 62.05, 61.98, 61.91, 27.03, 25.66 ppm; **MS (ESI)** m/z = 368.1792; $[M+Na]^+$ (calculated: $C_{12}H_{27}N_3NaO_6^+$: 368.1701).

Synthesis of compound 10

The amino derivative was synthesized by the reduction of compound 9 (1 g) where it was dissolved in methanol (4 mL), and further Pd/C (10%) was added to the vial. The whole reaction was stirred under an H_2 reactor (5 bar) for 24 h. After the completion of the reaction, the Pd/C was filtered, and the methanol was concentrated to yield the desired compound 10 in 95 % yield.

^{1}H NMR (500 MHz, MeOD) δ 4.34 – 4.17 (m, 2H), 4.05 (m, 2H), 3.72 (m, 2H), 3.62 – 3.34 (m, 8H), 3.08 (m, 1H), 1.38 (s, 6H), 1.33 (s, 6H) ppm; **^{13}C NMR** (176 MHz, MeOD) δ 110.51, 76.15, 74.00, 73.95, 73.30, 72.64, 67.61, 67.49, 56.01, 51.80, 51.77, 51.74, 27.06, 25.65 ppm; **MS (ESI)** m/z = 342.1887; $[M+Na]^+$ (calculated: $C_{12}H_{29}NNaO_6^+$: 342.1826).

<u>Synthesis of dTG-ene</u>

Sodium hydride (2.5 eq per OH-group) was dissolved under Argon conditions and ice cooling in THF (200 mL). Solketal (2.1 eq) and catalytic amounts of 15-Crown-5, potassium iodide and 18-crown-6 were added to the solution. 1,1-Bis(chloromethyl)ethylene (1.0 eq) was added slowly to the reaction mixture. The reaction was carried out at 65 °C overnight. The progress of the reaction was monitored by thin layer chromatography. After the reaction was completed, the THF was removed under reduced pressure and the crude was three times extracted using water/DCM. The combined organic layers were dried over Na_2SO_4 and concentrated. The product was purified by column chromatography using hexane/EtOAc. The three different stereoisomers were obtained with a yield of 82%.

^{1}H NMR (600 MHz, MeOD) δ 5.22 – 5.17 (m, 2H), 4.28 – 4.24 (m, 2H), 4.09 – 4.01 (m, 6H), 3.74 – 3.71 (m, 2H), 3.48 (qd, *J* = 10.1, 5.3 Hz, 4H), 1.38 (s, 6H), 1.33 (s, 6H) ppm. **^{13}C NMR** (151 MHz, MeOD) δ 144.12, 114.70, 110.46, 110.43, 96.86, 76.11, 76.04, 72.89, 72.15, 69.73, 67.55, 27.06, 25.69, 25.66 ppm. **MS (ESI)** m/z = 339.1785; $[M+Na]^+$ (calcd: $C_{18}H_{28}NaO_6^+$: 339.1886).

Synthesis of linear and dendritic detergents

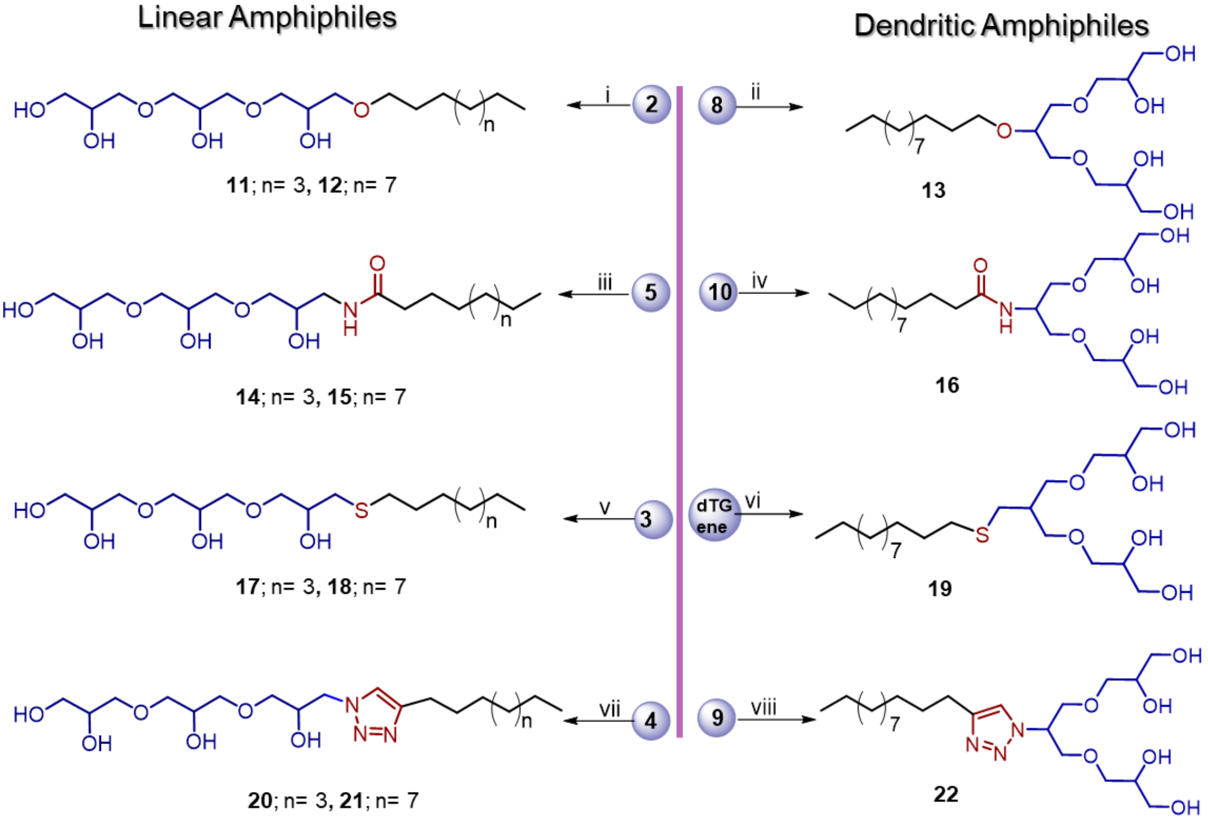

Supplementary Scheme 3. Synthesis of final detergents: i) a. Octanol/dodecanol, KOH, TBAB, Toluene, 48 h, 60 °C, b. Dowex-50, MeOH, 24 h, 50 °C; ii) Bromododecane, NaH, THF, 24 h, 60 °C, b. Dwoex-50, MeOH; iii)a Nonoic acid/ tridecanoic acid, EDC·HCl, HOBt, DCM, rt, 24 h, b. Dwoex-50, MeOH; iv) Nonoic acid, EDC·HCl, HOBt, DCM, rt, 24 h, b. Dwoex-50, MeOH; v)a. 20% HCL in MeOH, rt, 6 h, b. 1-dodecene/1-octene, DMPA, MeOH, LED 370 nm, 6 h, rt, vi) dodecanethiol, DMPA, MeOH, LED 370 nm, 6 h, rt, b. Dwoex-50, MeOH; vii) 1-decyne/1-tetradecyne, sodium ascorbate, copper sulphate, THF: Water (3:1), 24 h, 50 °C, b. Dwoex-50, MeOH; viii) 1-tetradeyne, sodium ascorbate, copper sulphate, THF: Water (3:1), 24 h, 50 °C, b. Dwoex-50, MeOH.

Synthesis of compounds 11 and 12

The linear detergents LTG-ether-C8 (**11**) and LTG-ether-C12 (**12**) with an ether spacer were synthesized *via* the opening of the epoxide ring where the Compound **2** (1 g, 1 eq) was dissolved in toluene (15 mL) followed by the addition of octanol (0.7 g, 1.5eq)/dodecanol (1 g, 1.5 eq). Further KOH (0.51 g, 1.2 eq) was added as base and TBAB (0.12 g, 0.1 eq) as a phase transfer catalyst. The whole reaction was left stirring for 24 h at 50 °C. After the completion of the reaction, toluene was removed under reduced pressure and then obtained

residue was extracted with water and DCM (30 mL x 3). The organic layer was dried over sodium sulphate and concentrated under reduced pressure. In the next step the obtained crude was subjected for deprotection of acetal group using mild acid to achieve the detergents **11** (52%) and **12** (56%), respectively.

(11) ^{1}**H NMR** (700 MHz, MeOD) δ 3.91 – 3.86 (m, 2H), 3.78 – 3.75 (m, 1H), 3.58 – 3.52 (m, 6H), 3.50 – 3.45 (m, 7H), 3.44 – 3.41 (m, 1H), 1.59 – 1.55 (m, 2H), 1.37 – 1.30 (m, 10H), 0.90 (t, *J* = 7.1 Hz, 3H) ppm; ^{13}**C NMR** (176 MHz, MeOD) δ 74.14, 74.11, 73.89, 73.86, 73.14, 72.62, 72.24, 72.20, 70.74, 70.70, 70.66, 64.32, 33.00, 30.71, 30.56, 30.42, 27.22, 23.71, 14.43 ppm; **MS (ESI)** m/z = 375.2559; $[M+Na]^+$ (calculated: $C_{17}H_{36}NaO_7^+$375.2353).

(12) ^{1}**H NMR** (700 MHz, MeOD) δ 3.92 – 3.85 (m, 2H), 3.78 – 3.75 (m, 1H), 3.59 – 3.52 (m, 6H), 3.50 – 3.44 (m, 7H), 3.44 – 3.41 (m, 1H), 1.59 – 1.55 (m, 2H), 1.37 – 1.29 (m, 18H), 0.90 (t, *J* = 7.1 Hz, 3H) ppm; ^{13}**C NMR** (176 MHz, MeOD) δ 74.15, 74.12, 73.90, 73.87, 73.15, 72.64, 72.25, 72.21, 70.75, 70.71, 70.68, 64.33, 33.07, 30.78, 30.75, 30.72, 30.60, 30.47, 27.22, 23.73, 14.44 ppm; **MS (ESI)** m/z = 431.3107; $[M+Na]^+$ (calculated: $C_{21}H_{44}NaO_7^+$: 431.2979).

Synthesis of compound 13

The dendritic detergent DTG-ether-C12 (**13**) was obtained via the substitution reaction where compound 8 (1 g, 1 eq) was dissolved in THF (15 mL), further a disperse solution of NaH (0.15 g, 2 eq) was added. The reaction flask stirred at rt for 30 minutes and then n-bromododecane (1.1 g, 1.3 eq) was added slowly. The combined reaction flask was stirred at 50 °C for 24 h. After the completion of the reaction, toluene was removed under reduced pressure and then obtained residue was extracted with water and DCM (30 mL x 3). The organic layer was dried over sodium sulphate and concentrated under reduced pressure. In the next step the obtained crude was subjected for deprotection of acetal group using mild acid to achieve the detergents **13** (75%).

^{1}**H NMR** (700 MHz, MeOD) δ 3.77 – 3.74 (m, 2H), 3.63 – 3.53 (m, 13H), 3.49 – 4.46 (m, 2H), 1.59 – 1.54 (m, 2H), 1.36 – 1.30 (m, 18H), 0.90 (t, *J* = 7.1 Hz, 3H) ppm; ^{13}**C NMR** (176 MHz, MeOD) δ 79.16, 73.97, 73.90, 72.18, 72.13, 71.52, 64.45, 33.06, 31.10, 30.78, 30.74, 30.60, 30.46, 27.18, 23.72, 14.46 ppm; **MS (ESI)** m/z = 431.3128; $[M+Na]^+$ (calculated: $C_{21}H_{44}NaO_7$: 431.2979).

Synthesis of compound 14 and 15

The detergents with amide linkage were synthesized using the acid/amine coupling mechanism in which protected LTG-NH_2 (**5**) (1 g, 1 eq), commercially available nonanoic acid (0.7 g, 1.3 eq)/tridecanoic acid (0.9 g, 1.3 eq) were dissolved in DCM (20 mL). Further, EDC·HCl (0.8 g, 1.2 eq) and HOBt (0.4 g, 1 eq) were added to the reaction flask. The whole

reaction flask was stirred at rt for the next 24 h. After the completion of the reaction, the reaction mixture was extracted with water and DCM (30 mL x 3). The organic layer was dried over sodium sulphate and concentrated under reduced pressure. In the next step the obtained crude was subjected for deprotection of acetal group using mild acid to achieve the detergents **14** (62%) and **15** (63%), respectively.

(14) ^{1}**H NMR** (600 MHz, METHANOL-*D*$_4$) δ 3.92 – 3.89 (m, 1H), 3.83 – 3.75 (m, 2H), 3.59 – 3.41 (m, 11H), 3.36 – 3.33 (m, 1H), 3.22 (dd, *J* = 13.7, 6.7 Hz, 1H), 2.21 – 2.19 (m, 2H), 1.63 – 1.58 (m, 2H), 1.33 – 1.29 (m, 18H), 0.90 (t, *J* = 7.1 Hz, 3H) ppm; ^{13}**C NMR** (151 MHz, METHANOL-*D*$_4$) δ 176.71, 74.59, 73.87, 73.83, 72.23, 72.20, 70.68, 70.37, 70.34, 64.31, 43.43, 37.07, 32.99, 30.43, 30.33, 27.01, 23.70, 14.43 ppm; **MS (ESI)** m/z = 402.2462; $[M+Na]^+$ (calculated: $C_{18}H_{37}NNaO_7^+$: 402.2618).

(15) ^{1}**H NMR** (700 MHz, MeOD) δ 3.92 – 3.89 (m, 1H), 3.83 – 3.75 (m, 2H), 3.59 – 3.41 (m, 10H), 3.36 – 3.33 (m, 1H), 3.22 (dd, *J* = 13.7, 6.7 Hz, 1H), 2.21 – 2.19 (m, 2H), 1.62 – 1.59 (m, 2H), 1.36 – 1.24 (m, 18H), 0.90 (t, *J* = 7.1 Hz, 3H) ppm; ^{13}**C NMR** (176 MHz, MeOD) δ 176.72, 74.61, 73.89, 73.84, 72.25, 72.21, 70.69, 70.37, 64.32, 43.45, 37.08, 33.07, 30.77, 30.74, 30.64, 30.46, 30.33, 27.02, 23.72, 14.43 ppm; **MS (ESI)** m/z = 458.3214; $[M+Na]^+$ (calculated: $C_{22}H_{45}NNaO_7^+$: 458.3088).

<u>Synthesis of compound 16</u>

DTG-NH_2 (**10**) (1 g, 1 eq) and tridecanoic acid (0.87 g, 1.3 eq) were dissolved in DCM (20mL) followed bz the addition of EDC·HCl (0.74 g, 1.2 eq) and HOBt (0.42 g, 1 eq). The combined reaction mixture was stirred at rt for the next 24 h. After the completion of the reaction, the reaction mixture was extracted with water and DCM (30 mL x 3). The organic layer was dried over sodium sulphate and concentrated under reduced pressure. In the next step the obtained crude was subjected for deprotection of acetal group using mild acid to achieve the detergents **16** (67%).

^{1}**H NMR** (700 MHz, MeOD) δ 4.19 – 4.16 (m, 1H), 3.78 – 3.73 (m, 2H), 3.59 – 3.44 (m, 12H), 2.20 (t, *J* = 7.5 Hz, 2H), 1.61 (t, *J* = 7.4 Hz, 2H), 1.33 – 1.28 (m, 17H), 0.90 (t, *J* = 7.1 Hz, 3H) ppm; ^{13}**C NMR** (176 MHz, MeOD) δ 176.27, 73.78, 73.53, 72.24, 72.16, 71.22, 71.13, 64.36, 64.33, 50.20, 37.10, 33.07, 30.76, 30.64, 30.47, 30.30, 27.07, 23.73, 14.43 ppm; **MS (ESI)** m/z = 458.3243; $[M+Na]^+$ (calculated: $C_{22}H_{45}NNaO_7^+$: 458.3088).

<u>Synthesis of compound 17 and 18</u>

The synthesis of LTG-thioether-C8 and LTG-thioether-C12 were synthesized using thio-ene click approached. For this, initially the protected LTG-SOAc (**3**) was stirred for 6 h with 20 % HCl in MeOH and then the solvent was removed under reduced pressure the obtained crude

which further react with 1- octene (1.2 eq) and 1-dodecene (1.2 eq) using DMPA (0.1 eq) as photoinitiator, and methanol as solvent and LED lamp (370 nm) for 6 h at rt. After the completion of the reaction, the methanol was removed under reduced pressure. The obtained crude further purified with RPHPLC to achieve the detergents **17**(62%) and **18** (63%), respectively.

(17) ^{1}**H NMR** (700 MHz, MeOD) δ 3.92 – 3.89 (m, 1H), 3.85 – 3.82 (m, 1H), 3.78 – 3.75 (m, 1H), 3.59 – 3.46 (m, 11H), 2.69 – 2.66 (m, 1H), 2.61 – 2.55 (m, 3H), 1.61 – 1.56 (m, 2H), 1.42 – 1.38 (m, 2H), 1.35 – 1.26 (m, 8H), 0.90 (t, *J* = 7.1 Hz, 3H) ppm; ^{13}**C NMR** (176 MHz, MeOD) δ 75.31, 75.28, 73.89, 73.86, 73.82, 72.24, 72.21, 71.35, 71.32, 70.72, 70.68, 64.33, 36.28, 33.69, 32.99, 30.84, 30.36, 30.32, 29.88, 23.70, 14.43 ppm; **MS (ESI)** m/z = 395.2325; $[M+Na]^+$ (calculated: $C_{17}H_{36}NaO_6S^+$: 391.2125).

(18) ^{1}**H NMR** (700 MHz, MeOD) δ 3.92 – 3.89 (m, 1H), 3.85 – 3.82 (m, 1H), 3.78 – 3.75 (m, 1H), 3.59 – 3.46 (m, 10H), 2.7 – 2.66 (m, 1H), 2.59 – 2.55 (m, 3H), 1.61 – 1.57 (m, 2H), 1.42 – 1.38 (m, 2H), 1.34 – 1.26 (m, 16H), 0.90 (t, *J* = 7.1 Hz, 3H) ppm; ^{13}**C NMR** (176 MHz, MeOD) δ 75.31, 75.29, 73.90, 73.87, 73.83, 72.25, 72.21, 71.36, 71.32, 70.72, 70.68, 64.33, 36.28, 33.70, 33.07, 30.85, 30.77, 30.73, 30.68, 30.47, 30.36, 29.88, 23.73, 14.45 ppm; **MS (ESI)** m/z = 461.3020; $[M+Na]^+$ (calculated: $C_{21}H_{44}NaO_6S^+$: 461.2907).

Synthesis of compound 19

DTG-thioether-C12 was obtained from protected dTG-ene (**Supplementary Scheme 2**), which was coupled with dodecane thiol with thio-ene click chemistry. The dTG-ene (1.5 g, 1 eq) was dissolved in methanol, followed by the dodecane thiol (0.7 g, 1.2 eq, DMPA (0. g, 0.1 eq) as photoinitiator the whole reaction mixture was radiated with LED lamp (370 nm) for 6h at rt. After the completion of the reaction, the methanol was removed under reduced pressure. The obtained crude further purified with RP-HPLC to achieve the detergents **19** in 95% yield.

^{1}**H NMR** (700 MHz, MeOD) δ 3.77 – 3.74 (m, 2H), 3.59 – 3.48 (m, 10H), 3.45 – 3.42 (m, 2H), 2.60 (d, *J* = 6.7 Hz, 2H), 2.51 (t, *J* = 7.3 Hz, 2H), 2.08 – 2.04 (m, 1H), 1.60 – 1.56 (m, 2H), 1.42 – 1.38 (m, 2H), 1.34 – 1.27 (m, 16H), 0.90 (t, *J* = 7.1 Hz, 3H) ppm; ^{13}**C NMR** (176 MHz, MeOD) δ 73.57, 72.20, 72.07, 64.56, 41.21, 33.64, 33.07, 32.02, 30.79, 30.75, 30.72, 30.69, 30.47, 30.37, 29.89, 23.73, 14.46 ppm; **MS (ESI)** m/z = 461.3040.6370; $[M+Na]^+$ (calculated: $C_{21}H_{44}NaO_6S^+$: 461.2907).

Synthesis of compounds 20 and 21

The detergents were obtained using the azide-alkyne click chemistry approach. The protected LTG-N_3 (**4**) (1 g, 1 eq) and 1-decyne (0.5 g, 1.2 eq)/1-tetradeyne (0.7 g, 1.2 eq), followed by the addition of copper sulphate (0.16 g, 0.2 eq) and sodium ascorbate (0.25g, 0.4eq) the whole

reaction mixture was dissolved in 3:1 mixture of THF/water. The combined reaction flask was heated at 50 °C for 24 h. After the completion of the reaction, solvent was removed under reduced pressure and then obtained residue was extracted with water and DCM (30 mL x 3). The organic layer was dried over sodium sulphate and concentrated under reduced pressure. In the next step the obtained crude was subjected for deprotection of acetal group using mild acid followed by purified with RP-HPLC to achieve the detergents LTG-triazole-C8 (**20**, 70%) and LTG-triazole-C12 (**21**, 72%),

(**20**) **^{1}H NMR** (700 MHz, MeOD) δ 7.63 (s, 1H), 4.45-4.43 (m, 1H), 4.31-4.28 (m, 1H), 4.02-4.00 (m, 1H), 3.84-3.81 (m, 1H), 3.68-3.64 (m, 1H), 3.47-3.36 (m, 10H), 2.59 (t, 2H), 1.59-1.55 (m, 2H), 1.28-1.18 (m, 10H), 0.80 (t, 3H) ppm; **^{13}C NMR** (176 MHz, MeOD) δ 147.63, 122.90, 72.41, 70.85, 69.15, 68.88, 62.93, 52.67, 31.61, 29.19, 29.03, 28.95, 28.85, 24.87, 22.30, 13.02 ppm; **MS (ESI)** m/z = 426.2740; $[M+Na]^+$ (calcd: $C_{19}H_{37}N_3NaO_6^+$: 426.2575).

(21) **^{1}H NMR** (600 MHz, MeOD) δ 7.70 (s, 1H), 4.53-4.49 (m, 1H), 4.37-4.34 (m, 1H), 4.10-4.07 (m, 1h), 3.90-3.88 (m, 1H), 3.75-3.71 (m, 1H), 3.55-3.42 (m, 10H), 2.66 (t, 2H), 2.65, 1.67-1.62 (m, 2H), 1.33-1.26 (m, 18H), 0.87 (t, 3H)ppm; **^{13}CNMR** (151 MHz,) δ 147.68, 122.97, 72.60, 72.46, 70.91, 69.34, 68.94, 62.98, 52.72, 31.73, 29.45, 29.35, 29.27, 28.92, 28.75, 24.94, 22.40, 12.99ppm; **MS (ESI)** m/z = 459.6280; $[M+Na]^+$ (calculated: $C_{23}H_{45}N_3NaO_6^+$: 482.3334).

<u>Synthesis of compound 22</u>

The protected DTG-N_3 (1 g, 1 eq) and 1-tetradeyne (0.6 g, 1.2 eq) were dissolved in (3:1) THF and water (10mL), copper sulphate (0.14 g, 0.2 eq) and sodium ascorbate (0.,22 g, 0.4 eq) was added to the reaction flask. The combined reaction flask was heated at 50 °C for 24 h. After the completion of the reaction, solvent was removed under reduced pressure and then obtained residue was extracted with water and DCM (30 mL x 3). The organic layer was dried over sodium sulphate and concentrated under reduced pressure. In the next step the obtained crude was subjected for deprotection of acetal group using mild acid followed by purified with RP-HPLC to achieve the DTG-triazole-C12 (**22**, 82%)

^{1}H NMR (700 MHz, MeOD) δ 7.89 (s, 1H), 4.98 – 4.95 (m, 1H), 3.97 – 3.90 (m, 4H), 3.72 – 3.69 (m, 2H), 3.55 – 3.43 (m, 8H), 2.69 (t, *J* = 7.7 Hz, 2H), 1.70 – 1.65 (m, 2H), 1.39 – 1.27 (m, 18H), 0.90 (t, *J* = 7.1 Hz, 3H) ppm; **^{13}C NMR** (176 MHz, MeOD) δ 148.80, 123.30, 73.79, 73.69, 72.13, 72.06, 71.31, 71.28, 64.25, 64.23, 62.37, 33.06, 30.77, 30.68, 30.55, 30.46, 30.30, 26.27, 23.72, 14.43 ppm; **MS (ESI)** m/z = 482.3351; $[M+Na]^+$ (calculated: $C_{23}H_{45}N_3NaO_6^+$: 482.3201).

4. Supplementary References

(1) Urner, L. H.; Junge, F.; Fiorentino, F.; El-Baba, T. J.; Shutin, D.; Nölte, G.; Haag, R.; Robinson, C. V. Rationalizing the Optimization of Detergents for Membrane Protein Purification. *Chem. Eur. J.* **2023**, *29*, e202300159.

(2) Urner, L. H.; Liko, I.; Yen, H.-Y.; Hoi, K. K.; Bolla, J. R.; Gault, J.; Almeida, F. G.; Schweder, M.-P.; Shutin, D.; Ehrmann, S.; et al. Modular detergents tailor the purification and structural analysis of membrane proteins including G-protein coupled receptors. *Nat. Commun.* **2020**, *11*, 564.
