# Supplementary material for: Chemical linkers switch triglycerol detergents from bacterial protein purification to mild antibiotic amplification
Source: Commun Chem. 2025 Mar 8;8:70. doi: 10.1038/s42004-025-01477-3 (PMC11890857; doi:10.1038/s42004-025-01477-3)
Supplement: Supplementary file 3 — Description of Additional Supplementary Files [file 42004_2025_1477_MOESM3_ESM.pdf]

Description of Additional Supplementary Files

File name: Supplementary Data 1

Description: Source Data
