# Supplementary material for: Chemical linkers switch triglycerol detergents from bacterial protein purification to mild antibiotic amplification
Source: Commun Chem. 2025 Mar 8;8:70. doi: 10.1038/s42004-025-01477-3 (PMC11890857; doi:10.1038/s42004-025-01477-3)
Supplement: Supplementary file 5 — Reporting Summary [file 42004_2025_1477_MOESM5_ESM.pdf]

nature portfolio

Corresponding author(s): Abhishek K. Singh, Leonhard H. Urner

Last updated by author(s): 22.02.2025

Reporting Summary

Nature Portfolio wishes to improve the reproducibility of the work that we publish. This form provides structure for consistency and transparency in reporting. For further information on Nature Portfolio policies, see our Editorial Policies and the Editorial Policy Checklist.

Statistics

For all statistical analyses, confirm that the following items are present in the figure legend, table legend, main text, or Methods section.

n/a	Confirmed	
☐	☒	The exact sample size (*n*) for each experimental group/condition, given as a discrete number and unit of measurement
☐	☒	A statement on whether measurements were taken from distinct samples or whether the same sample was measured repeatedly
☐	☒	The statistical test(s) used AND whether they are one- or two-sided<br>*Only common tests should be described solely by name; describe more complex techniques in the Methods section.*
☒	☐	A description of all covariates tested
☐	☒	A description of any assumptions or corrections, such as tests of normality and adjustment for multiple comparisons
☒	☐	A full description of the statistical parameters including central tendency (e.g. means) or other basic estimates (e.g. regression coefficient) AND variation (e.g. standard deviation) or associated estimates of uncertainty (e.g. confidence intervals)
☒	☐	For null hypothesis testing, the test statistic (e.g. *F*, *t*, *r*) with confidence intervals, effect sizes, degrees of freedom and *P* value noted<br>*Give P values as exact values whenever suitable.*
☒	☐	For Bayesian analysis, information on the choice of priors and Markov chain Monte Carlo settings
☒	☐	For hierarchical and complex designs, identification of the appropriate level for tests and full reporting of outcomes
☒	☐	Estimates of effect sizes (e.g. Cohen's *d*, Pearson's *r*), indicating how they were calculated

Our web collection on statistics for biologists contains articles on many of the points above.

Software and code

Policy information about availability of computer code

Data collection	SDS PAGE data were collected with a ChemiDoc MP Imaging System software (BIORAD), analytical HPLC chromatograms wre collected with ClarityChrom version 8.5.0.64 (Knauer), UV/Vis data were collected with the NanoPhotometer(R) NP80 software (IMPLEN)
Data analysis	NMR data were analyzed and visualized with MestreNova version 6.0.2-5475; critical micelle concentrations, relative protein quantities, HLB values, analytical HPLC chromatograms, and antibiotic amplification factors were calculated and/or visualized with OriginPro 2024b version 10.1.5.132 (OriginLab); chemical structures were drawn with ChemDraw professional version 23.1.1.3 in ACS document 1996 style, data visualized in OriginPro and Chemdraw were exported as ai.files or directly copied to finalize images for publication in Adobe Illustrator CS2 version 12.0.0.

For manuscripts utilizing custom algorithms or software that are central to the research but not yet described in published literature, software must be made available to editors and reviewers. We strongly encourage code deposition in a community repository (e.g. GitHub). See the Nature Portfolio guidelines for submitting code & software for further information.

Data

Policy information about availability of data

All manuscripts must include a data availability statement. This statement should provide the following information, where applicable:

- Accession codes, unique identifiers, or web links for publicly available datasets
- A description of any restrictions on data availability
- For clinical datasets or third party data, please ensure that the statement adheres to our policy

Datasets generated during and/or analysed during the current study are available from the corresponding authors on reasonable request.

Research involving human participants, their data, or biological material

Policy information about studies with human participants or human data. See also policy information about sex, gender (identity/presentation), and sexual orientation and race, ethnicity and racism.

Reporting on sex and gender	not applicable in this study
Reporting on race, ethnicity, or other socially relevant groupings	not applicable in this study
Population characteristics	not applicable in this study
Recruitment	not applicable in this study
Ethics oversight	not applicable in this study

Note that full information on the approval of the study protocol must also be provided in the manuscript.

Field-specific reporting

Please select the one below that is the best fit for your research. If you are not sure, read the appropriate sections before making your selection.

☒ Life sciences ☐ Behavioural & social sciences ☐ Ecological, evolutionary & environmental sciences

For a reference copy of the document with all sections, see nature.com/documents/nr-reporting-summary-flat.pdf

Life sciences study design

All studies must disclose on these points even when the disclosure is negative.

Sample size	No sample-size calculation was performed. The applied host system was E. coli which was used for membrane protein expression or antibiotic amplification experiments. We followed previously published protein expression protocols and isolated the membranes of E. coli as stated within the Methods section of our manuscript. We also followed previously published antibiotic amplification experiments as stated within the Methods section of our manuscript.
Data exclusions	No data were excluded from the analysis.
Replication	We confirm that experiments were replicated and all attempts at replication were successful.
Randomization	We tested all our detergents systematically rendering randomization not necessary in our experiments.
Blinding	Our experiments did not involve subjective trials rendering blindings not necessary in our experiments.

Reporting for specific materials, systems and methods

We require information from authors about some types of materials, experimental systems and methods used in many studies. Here, indicate whether each material, system or method listed is relevant to your study. If you are not sure if a list item applies to your research, read the appropriate section before selecting a response.

Materials & experimental systems

n/a	Involved in the study	
☒	☐	Antibodies
☒	☐	Eukaryotic cell lines
☒	☐	Palaeontology and archaeology
☒	☐	Animals and other organisms
☒	☐	Clinical data
☒	☐	Dual use research of concern
☒	☐	Plants

Methods

n/a	Involved in the study	
☒	☐	ChIP-seq
☒	☐	Flow cytometry
☒	☐	MRI-based neuroimaging

Plants

Seed stocks	The study did not involve seed stocks.
Novel plant genotypes	The study did not involve novel plant genotypes.
Authentication	Authentication was not applicable in this study.
